# The feasibility and stability of large complex biological networks: a random matrix approach

**DOI:** 10.1038/s41598-018-26486-2

**Published:** 2018-05-29

**Authors:** Lewi Stone

**Affiliations:** 1 Biomathematics Unit, Faculty of Life Sciences, Tel Aviv University, Ramat Aviv, Israel; 2 Mathematical Sciences, Faculty of Science, RMIT University, Melbourne, Australia

## Abstract

In the 70’s, Robert May demonstrated that complexity creates instability in generic models of ecological networks having random interaction matrices **A**. Similar random matrix models have since been applied in many disciplines. Central to assessing stability is the “circular law” since it describes the eigenvalue distribution for an important class of random matrices **A**. However, despite widespread adoption, the “circular law” does not apply for ecological systems in which density-dependence operates (i.e., where a species growth is determined by its density). Instead one needs to study the far more complicated eigenvalue distribution of the community matrix **S** = **DA**, where **D** is a diagonal matrix of population equilibrium values. Here we obtain this eigenvalue distribution. We show that if the random matrix **A** is locally stable, the community matrix **S** **=** **DA** will also be locally stable, providing the system is feasible (i.e., all species have positive equilibria **D** > 0). This helps explain why, unusually, nearly all feasible systems studied here are locally stable. Large complex systems may thus be even more fragile than May predicted, given the difficulty of assembling a feasible system. It was also found that the degree of stability, or resilience of a system, depended on the minimum equilibrium population.

## Introduction

Network models have become indispensable tools for helping understand the biological processes responsible for the stability and sustainability of biological systems^1–21^. Intuitively, rich highly interconnected biological networks are expected to be the most stable, and are thus likely to better withstand the loss of a link, or to cope in the presence of external environmental perturbations. In the 70’s, May^1,2^ exploited random matrix theory (RMT), and the “circular law” for matrix eigenvalue distributions^22–24^, to challenge this paradigm. He demonstrated that more complex and connected ecological systems are in fact more fragile, and less likely to be stable in terms of their ability to recover after some small external perturbation. Since then, the RMT framework has proved extremely useful for identifying those factors that beget stability in large ecological communities^3–15^. Moreover, in recent years, the modeling approach has successfully spread to other disciplines, ranging from systems biology, neurosciences, through to atomic physics, wireless, finance and banking, making this an exciting and vibrant contemporary research discipline^16–21^.

Here I re-examine similar issues of stability versus complexity, while using a better suited formulation of a biological system’s “community matrix”–one that explicitly allows for the standard textbook assumption of density-dependent (DD) growth^2,25,26^. Such growth proves to be the rule rather than the exception for many biological processes, yet surprisingly, very little is known about their stability properties. In principle, May’s conclusions are not automatically translatable to DD systems. As we shall see, the “circular law” which sits at the foundation of May’s analysis, and governs the eigenvalue distribution of random matrices, generally does not hold for DD systems. The problem has resurfaced in recent prominent studies of ecological networks^6^.

In this paper we develop methods to predict eigenvalue distributions of large complex DD systems. In the process, the analysis results in new conclusions about the currently topical constraint of *feasibility*^6,7,10,15^. Feasibility requires that all equilibrium populations of a system are positive, a characteristic feature that is generally to be expected for any persistent system. There have been numerous reports in the literature of a strong association between feasibility of DD systems and stability, similar to Roberts^7^ who found that almost all feasible model systems are stable (see also refs^10–12^). The paper will be examining this association.

## Robert May’s “Neutral Interaction” Model of Large Complex Systems

It is helpful to first recall the original argument of May^1^. For an *n*-species community, let *N*_*i*_(*t*) be the abundance or biomass of the *i*’th species and let ${N}_{i}^{\ast }$ be the species equilibrium value (the symbol * indicating equilibrium). The actual units of *N*_*i*_(*t*) will depend on the details of the particular model and on many occasions it is convenient to scale the populations as we discuss shortly. We suppose that the growth rate of species-*i* depends on its interactions with other species as defined by some possibly complicated nonlinear function ${f}_{i}({N}_{1},{N}_{2},\ldots .\,,{N}_{n})$. That is:

1 $$\frac{d{N}_{i}}{dt}={f}_{i}({N}_{1},{N}_{2},\ldots .\,,{N}_{n})\,\,i=1,2,\ldots ,n.$$

Close to equilibrium, the abundance of species-*i* is given by ${N}_{i}(t)={N}_{i}^{\ast }+{x}_{i}(t)$, where *x*_*i*_(*t*) is the perturbation from the equilibrium value at time *t*. The dynamics of the population perturbations, when linearized around equilibrium, is of the form^1,2^:

2 $$\frac{d{\bf{x}}}{dt}={\bf{A}}\,{\bf{x}}.$$

The vector ${\bf{x}}=({x}_{i})$ contains the perturbed population disturbances *x*_*i*_(*t*). The matrix **A** is referred to as the “community matrix” or Jacobian matrix and has elements ${a}_{ij}={(\frac{\partial {f}_{i}}{\partial {N}_{j}})}^{\ast }.$ Here *a*_*ij*_ represents the effect species-*j* has on the growth of species-*i* when close to equilibrium. A cooperative effect implies ${a}_{ij} > 0$, while a negative effect is just the opposite with ${a}_{ij} < 0$. The self-interactions between species are all scaled such that ${a}_{ii}=-\,1$, as in May^1^.

May^1^ studied communities under the limited *“neutral interaction”* assumption where interspecific interactions are equally positive as negative, and their expected or average value is zero i.e., E(${a}_{ij}$) = 0. Environmental fluctuations are assumed to perturb the interaction strengths, so that the community matrix is:

3 $${\bf{A}}=-\,{\bf{I}}+{\bf{B}},$$

where **I** is the identity matrix. The matrix **B** is a random matrix with coefficients *b*_*ij*_ drawn from some random distribution having mean zero and variance Var(*b*_*ij*_) = *σ*^2^, while diagonal terms ${b}_{ii}=0.$ Entries of the matrix **B** are identically distributed independent random variables, and thus have no correlations. The variability of the interaction strengths *σ* reflects the strength of the disturbance perturbing the ecological system. Finally, to model connectance of the interaction network, a proportion $\,(1-C)$ of randomly chosen off-diagonal interactions *a*_*ij*_ are set to zero, leaving a proportion *C* nonzero.

We are interested in finding conditions for the “local stability” of these biological models which guarantee that a system will return to equilibrium after a “small” population perturbation. Unless otherwise stated, the paper will be concerned exclusively with local stability. It is well known that if all eigenvalues (*λ*_*i*_) of the community matrix **A** have negative real parts (*Re*(*λ*_*i*_) < 0), the system is locally stable. Thus local stability depends on the critical eigenvalue of the community matrix **A** that has the largest real part, i.e., on the stability threshold:

4 $${\rm{\Lambda }}={\max }_{i}\,Re({\lambda }_{i}).$$

The system is locally stable if Λ < 0, in which case all perturbations *x*_*i*_(*t*) eventually die out and the populations eventually converge to their equilibrium values ${N}_{i}^{\ast }$. When the eigenvalues of a matrix **A** are such that Λ < 0, it is sometimes convenient to refer to **A** as a “stable matrix.” Instability occurs when Λ > 0.

**Figure 1:**
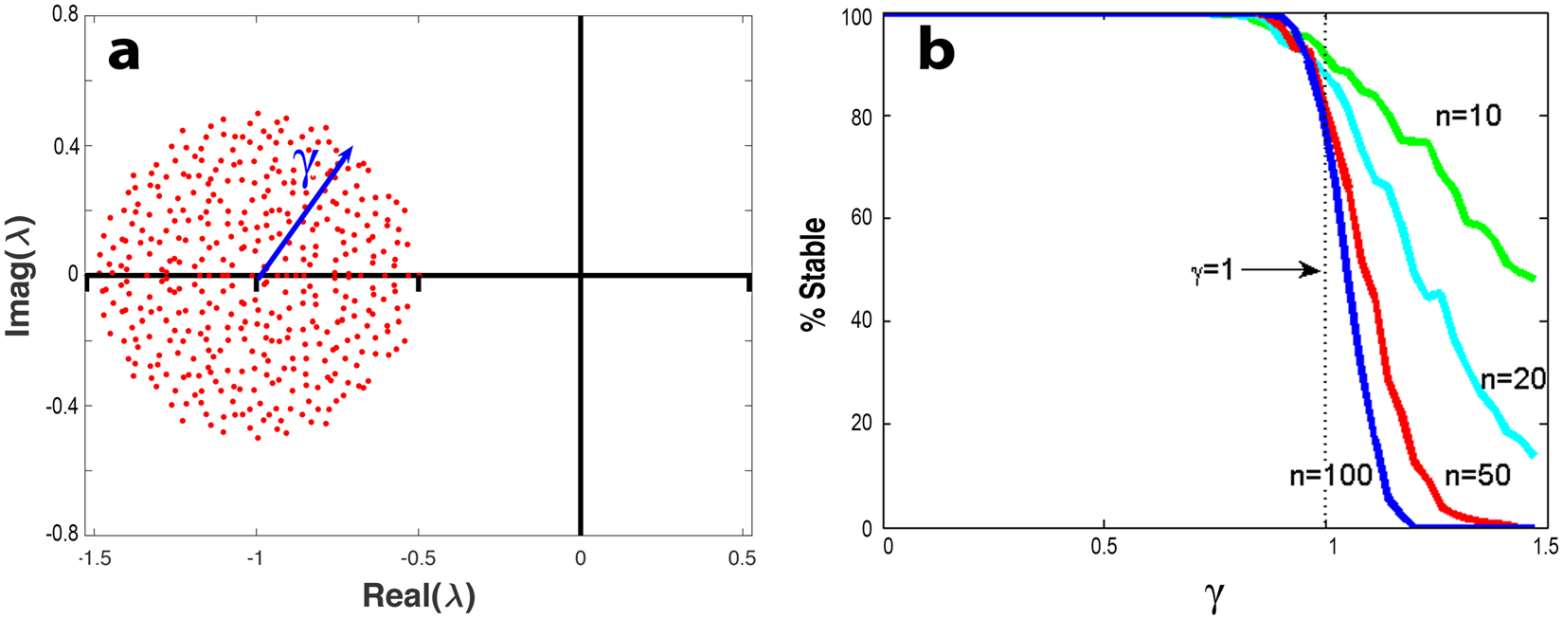
(**a**) The distribution of eigenvalues (red points) of the random matrix **A** = −**I** + **B** in the complex plane for *n* = 400 and $\gamma =\sqrt{n\,}\sigma =0.5$, as determined numerically. The eigenvalues fall in a circle having radius γ, centred at the point (−1, 0). When γ > 1, the eigenvalues enter the RHS of complex plane indicating instability. (**b**) Percentage of locally stable interaction matrices **A** as a function of disturbance *γ* in an ensemble of 500 matrices for different community-sizes *n* = *10*, *20*, *50*, *100*. May’s stability threshold sits at *γ* = 1. Classical results of May^1^ as published in Stone^10,11^.

A central result in RMT states that the eigenvalues of random matrices such as **A** are distributed according to a “circular law.” Specifically, the *n* eigenvalues *λ*_*i*_ of **A** are distributed uniformly in a circle with radius $\gamma =\sqrt{nC\,}\sigma$ in the complex plane^22–24^. As an example, Fig. 1a visualises the distribution of eigenvalues of the random matrix ${\bf{A}}=-\,{\bf{I}}+{\bf{B}}$ in the complex plane for *n* = 400 and $\gamma =\sqrt{n\,}\sigma =0.5$, as determined numerically. The eigenvalues clearly fall in a circle having radius γ. The circle is centred at the point (−1, 0) and thus translated one unit to the left of the origin (0, 0) as a result of the identity matrix −**I** that is present in **A**.

In this paper, it is often of interest to study the properties of each new matrix **A**, as *γ* is increased incrementally from zero. If the radius of the eigenvalues circular distribution is increased to the point where it exceeds *γ* = 1, then one or more eigenvalues of **A** will populate the right-hand-side (RHS) of the complex plane (see Fig. 1a). In that case, at least one eigenvalue has a real part that is positive, so that Λ > 0. But the latter is the aforementioned condition for triggering instability.

This was the argument used by May^1^ to demonstrate that Eq. 1 is locally stable for the neutral interaction model, if the interaction disturbances are “not too large,” namely if:

5 $${\boldsymbol{\gamma }}=\sqrt{nC\,}\sigma  < 1,$$

and unstable otherwise. The larger the number of species *n*, the sharper the transition from stability to instability at ${\boldsymbol{\gamma }}=1$. This is visualised in Fig. 1b which plots the percentage of random matrices that are locally stable as a function of disturbance ***γ***. In terms of model parameters, the threshold criterion Eq. 5 means that if either *n*, *σ* or *C* become too large, the system will transition into an unstable regime. With this simple but powerful argument, May demonstrated the fragility of large complex and highly connected systems.

## Eigenvalue Distribution of Density-Dependent Community Matrix **S** = **DA**

More plausible biological models that include the operation of density-dependence (DD), may be framed in the form^2,25,26^

6 $$\frac{d{N}_{i}}{dt}={N}_{i}\,{g}_{i}({N}_{1},{N}_{2},\ldots .\,,{N}_{n})\,\,i=1,2,\ldots ,n$$

In these models, the *net per-capita* growth rate of an *individual* of species-*i* (i.e. 1/*N*_*i*_ *dN*_*i*_/*dt*) depends on its interactions with other species as defined by some (often complicated) function ${g}_{i}({N}_{1},{N}_{2},\ldots .\,,{N}_{n})$. In the simplest DD model, $d{N}_{i}/dt=r\,{N}_{i}$, and each species has the same constant per-capita growth rate *r*, giving rise to exponential growth for each species. The well known Lotka-Volterra equations are a paradigmatic example of a more complex DD model, as discussed below.

We will examine whether or not a feasible equilibrium solution of Eq. (6), ${{\bf{N}}}^{\ast }=({N}_{1}^{\ast },\,{N}_{2}^{\ast },\ldots \,\ldots .{N}_{n}^{\ast })$, is stable. But stability of this equilibrium is no longer solely determined by eigenvalues of matrices of the form **A**, as defined earlier. Linearizing Eq. 6 about equilibrium using a Taylor expansion yields an equation for the perturbations^2^:

7 $$\frac{d{\bf{x}}}{dt}={\bf{D}}{\bf{A}}\,{\bf{x}}$$

where the diagonal matrix **D** = *diag*(${N}_{i}^{\ast }$). Stability of the perturbations is determined by the critical eigenvalue of the community matrix **S** = **DA**, where the diagonal matrix **D** = *diag*(${N}_{i}^{\ast }$)^2,19,20^.

Again, local stability of a feasible equilibrium is guaranteed if the critical eigenvalue component of **S** satisfies Λ(**S**) < 0. It is important to emphasise, that even though the matrix **A** might be stable, this does not automatically imply the matrix **S** = **DA** is stable (**D** > 0)^6–10^. Only very special classes of matrices have the property of D-stability for which stability of **A** implies **S** = **DA** is stable for any **D** > 0 (see ref.^27^ and below).

**Figure 2:**
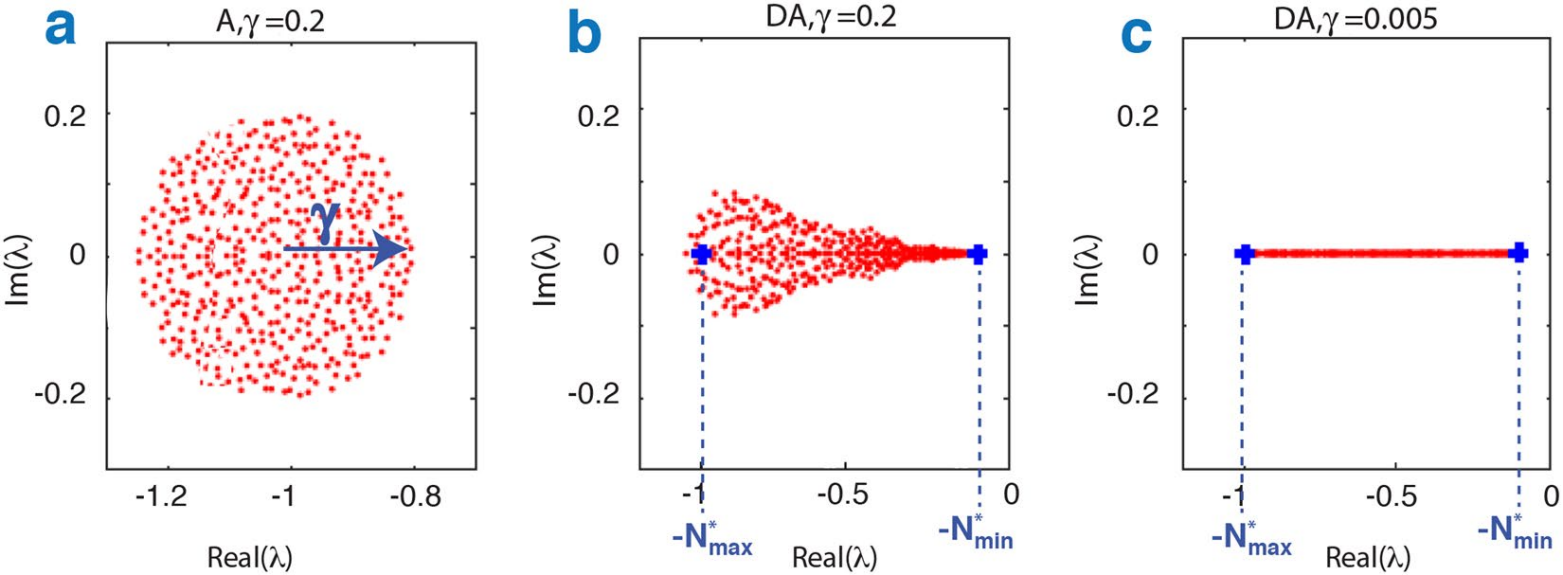
(**a**) Eigenvalues of the matrix **A** in the complex plane for *n* = 400, γ = 0.2 are distributed according to the “circular law” and fall in a circle centred at (−1, 0) having radius γ. (**b**) The eigenvalue distribution for the community matrix **S** = **DA**, where **D** = *diag(*${N}_{i}^{\ast }$*)* is a positive diagonal matrix with the same matrix **A** as in (**a**). The circular distribution disappears and is replaced by a “guitar-shaped” distribution in which the imaginary components of the eigenvalues appear flattened out compared with (**a**). The extreme left-hand and right-hand eigenvalues are predicted well by $\mbox{--}{N}_{max}^{\ast }$ and $\mbox{--}\,{N}_{min}^{\ast }$ (blue+). (**c**) Same as (**b**) but with γ = 0.01. Now nearly all eigenvalues are real and sit close to the real axis wedged between $[-{N}_{max}^{\ast },\,-{N}_{min}^{\ast }]$.

A useful although hypothetical starting point is to assume that all *n* population equilibria ${N}_{i}^{\ast }$are randomly distributed in the interval (0, 1), and then examine the community matrix **S** = **DA**, taking **A** as a random matrix (*n* = 400, γ = 0.2). While **A** has eigenvalues distributed in a circle in the complex plane as shown in Fig. 2a, this is no longer the case for the community matrix **S** = **DA** which now has a “guitar-shaped” distribution as seen in Fig. 2b. The eigenvalue distribution for the community matrix **S** = **DA** has the same matrix **A** as in Fig. 2a. The circular law for **A** becomes stretched and distorted as an outcome of the multiplication with the matrix of population densities **D** = *diag(*${N}_{i}^{\ast }$*)*.

In the extreme limiting case, when all off-diagonal interspecific interactions are set to zero (γ = 0), the eigenvalues have precisely the same magnitude as the equilibrium population values with ${\lambda }_{i}=-\,{N}_{i}^{\ast }$. Figure 2c shows a situation very near to this case with γ = 0.01, for which there are many weak off-diagonal interspecific interactions, and nearly all of the eigenvalues sit on the real axis in close proximity to the equilibrium population values ${\lambda }_{i}\simeq -\,{N}_{i}^{\ast }$. Thus, if we denote the smallest and largest equilibrium population as ${N}_{min}^{\ast }$ and ${N}_{max}^{\ast }$ (in blue), then all eigenvalues should be wedged in the interval $-[{N}_{max}^{\ast },\,{N}_{min}^{\ast }]$ in the complex plane as seen in Fig. 2c between the two demarked points in blue.

Here we show how to extract the eigenvalue distribution for **S** = **DA**. In a recent important paper in the context of neuronal networks, Ahmadian *et al*.^16^ studied the eigenvalue distribution of matrices having forms similar to the community matrix ${\bf{S}}={\bf{D}}{\bf{A}}={\bf{D}}(\,-\,{\bf{I}}+{\bf{B}})$ where **B** is a random matrix. Their results imply that for large *n*, the eigenvalue density of **S** is nonzero in the region of the complex plane, satisfying:

8 $${\rm{trace}}\,[{({{\bf{D}}}_{z}{{\bf{D}}}_{z}^{\dagger })}^{-1}]\ge 1/{\sigma }^{2}\,{\rm{where}}\,{{\bf{D}}}_{z}={{\bf{D}}}^{-1}(z{\bf{I}}-{\bf{D}}).$$

The complex variable $z=x+iy$, and trace(**A**) = ${\sum }^{}{a}_{ii}$ is defined as the usual sum of the matrix **A**’s diagonal elements. In the Methods section, it is shown that the region corresponds to those values of $z=x+iy$ for which:

9 $$T={\sum }_{i=1}^{n}\frac{{({N}_{i}^{\ast })}^{2}}{(z+{N}_{i}^{\ast })(\bar{z}+{N}_{i}^{\ast })}\ge 1/[C{\sigma }^{2}].$$

The inequality specifies a well-defined region in the complex plane where the eigenvalues of **S** lie. The region is referred to as the “support” of the eigenvalue distribution, and unlike the RMT circular law, the eigenvalue density is generally not uniform in this region. Note though, that a potential caveat of the method of Ahmadian *et al*.^16^ is that it depends on the matrices **D** and **A** being independent. But for our application the caveat appears to be relatively inconsequential (see SI6).

Inequality Eq. 9 shows that the support region of the eigenvalues is determined exclusively by the equilibrium populations ${N}_{i}^{\ast }$, σ, and connectance *C*. Furthermore, one immediately observes that *T* has singularities at those points where $z=-\,{N}_{i}^{\ast }$, indicating that the region containing the eigenvalues of **S** must necessarily envelope the population equilibria −${N}_{i}^{\ast }$. This gives an important hint of the strong relationship between the eigenvalues and the population equilibria.

**Figure 3:**
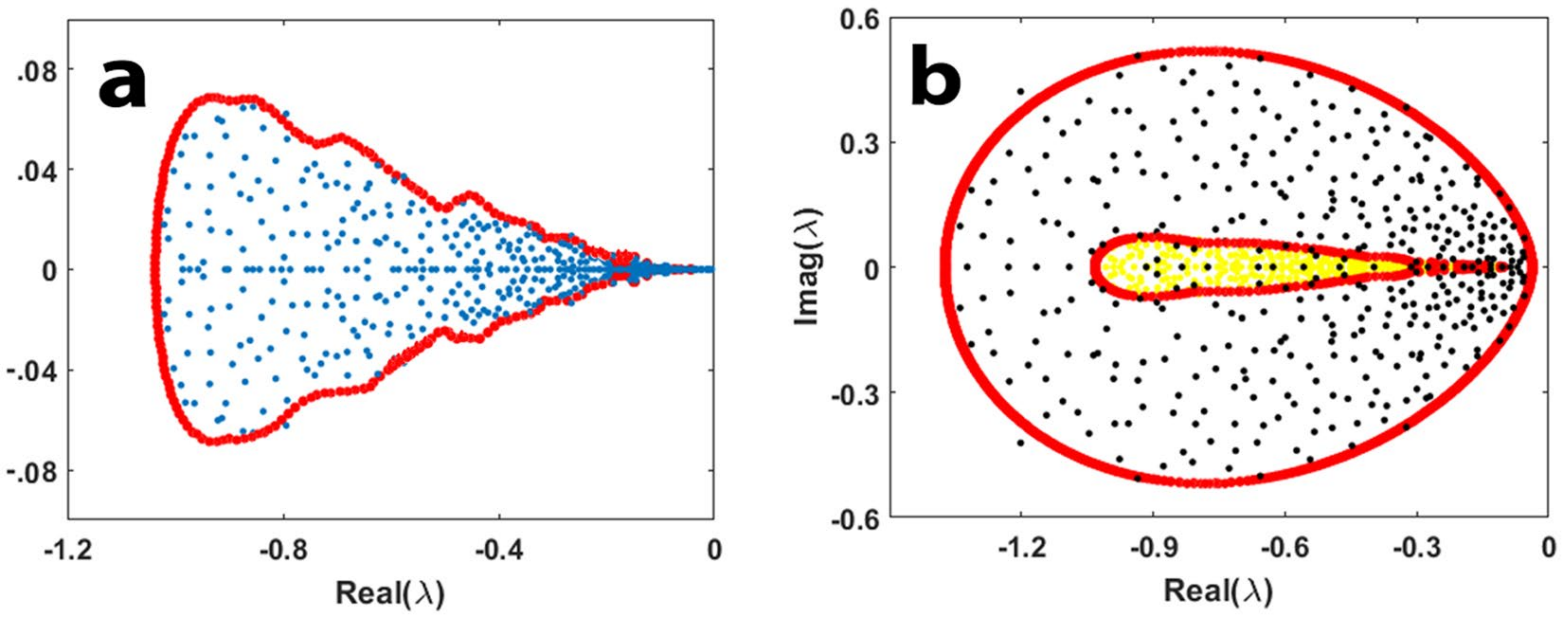
(**a**) Eigenvalues (blue dots) of community matrix **S** = **DA** distributed in the complex plane, where $\,n=400,\,\gamma =\,$0.2, **D** = *diag(*${N}_{i}^{\ast }$*)*, and ${N}_{i}^{\ast }$ are uniformly drawn from interval [0.05, 1]. Eigenvalue boundary appears as red dots, as obtained from Eq. 5 evaluated at equality. (**b**) Similar but with eigenvalues as yellow dots for *γ* = 0.2, and blue dots for *γ* = 0.9. The ${N}_{i}^{\ast }$ are uniformly drawn from interval [0.1, 1].

It is possible to capture the complicated eigenvalue boundary that arises by evaluating Eq. 9 at equality. Figure 3a plots the eigenvalue distribution for a typical community matrix **S** with *n* = *400*, σ = *0*.*01*, and *C* = 1 (*i*.*e*.,*γ* = 0.2), while the ${N}_{i}^{\ast }$ are chosen from a uniform distribution in the interval [0.05, 1]. The boundary indicated in red is the curve deduced from Eq. 9 evaluated at equality. Equation 9 accurately predicts the borders of the eigenvalue distribution, and envelopes all equilibrium populations: −${N}_{i}^{\ast }\in [\,-\,1,-\,0.05]$.

Figure 3b plots the eigenvalues of **S** for both *γ* = 0.2 (yellow dots) and *γ* = 0.9 (blue dots) superimposed on the same graph. Note that as *γ* is increased to *γ* = 0.9, the red boundary expands considerably. When *γ* > 1, the boundary moves into the RHS of the complex plane (not shown) where eigenvalues have positive real parts $(Re({\lambda }_{i}) > 0)$, and the system is unstable.

Finally, it is not hard to see from Eq. 9 that if we set all ${N}_{i}^{\ast }=1,$ the May model is retrieved, and all eigenvalues lie in a circle in the complex plane centred at the point $z=-\,1$, having radius $\,\gamma =\sqrt{nC}\sigma$. This of course retrieves May’s result Eq. 5, that stability is ensured if ***γ*** < 1.

For a given system, the eigenvalue distribution changes as $\gamma =\sqrt{nC}\sigma$ is increased, similar to that shown in Fig. 3b for two different values of *γ*. For small *γ*, the eigenvalues all sit in the LHS of the complex plane, and the system is stable (the critical eigenvalue component is Λ(**S**) = ${{\rm{\max }}}_{i}\,Re({\lambda }_{i}) < 0)$. As *γ* is increased beyond a threshold point, the eigenvalues start to populate the RHS and the system becomes unstable (Λ > 0). Based on Eq. 5, as *γ* is increased from zero, the threshold between stability and instability occurs when the right-most point of the elliptical-like eigenvalue boundary (red line) first touches the point *z* = 0 at the origin, in the complex plane. That is, where Λ(**S**) = ${{\rm{\max }}}_{i}\,Re({\lambda }_{i})=0.$ The threshold value of $\gamma$, can be found by evaluating Eq. 9 at equality with *z* = 0:

10 $$1={\sum }_{i=1}^{n}\frac{C{({N}_{i}^{\ast }\sigma )}^{2}}{(0-{N}_{i}^{\ast })(0-{N}_{i}^{\ast })}={\sum }_{i=1}^{n}\frac{C{({N}_{i}^{\ast }\sigma )}^{2}}{{({N}_{i}^{\ast })}^{2}}=nC{\sigma }^{2}={{\rm{\gamma }}}^{2}.$$

And thus the feasible equilibrium N^*^ is locally stable if:

11 $$\gamma =\sqrt{nC}\sigma  < 1,$$

which surprisingly is independent of the positive equilibrium populations. The system is unstable if $\gamma =\sqrt{nC}\sigma  > 1$. We thus find that May’s stability criterion is unusually general and holds for DD systems having community matrices of the form **S** = **DA**, even though the eigenvalue distributions of the latter are far from “circular.”

Note that the identical stability criteria (5) and (11) for **A** and **S** = **DA** are statistical criteria for an ensemble of matrices, and do not necessarily imply that the stability of the individual matrix **A** guarantees the stability of the matrix **S** = **DA**. However, based on the above results, it is demonstrated in SI2 that for these feasible RMT systems the matrices **A** and **S** = **DA** become unstable at exactly the same parameter values (approximately *γ* = 1). Thus for large feasible systems (**D** > 0):

12 $$\begin{array}{c}{\rm{stability}}\,{\rm{of}}\,{\rm{the}}\,{\rm{interaction}}\,{\rm{matrix}}\,{\bf{A}}\,{\rm{implies}}\\ {\rm{stability}}\,{\rm{of}}\,{\rm{the}}\,{\rm{community}}\,{\rm{matrix}}\,{\bf{S}}={\bf{D}}{\bf{A}}.\end{array}$$

where **A** is a random matrix as defined by May^1^. This is similar but not exactly the same as D-stability *(6*,*11*,*27)*, where by definition local stability of the interaction matrix **A** implies local stability of the community matrix **S** = **DA**, for *any* **D** > 0.

It is important to emphasise that our results concerning the eigenvalue distribution of the stability matrix **S** = **DA** here, depend on the work of Ahmadian *et al*.^16^, and thus assume that the matrix **D** is fixed and deterministic while the matrix **A** is random. This is not always the case, as will be discussed when we study the Lotka-Volterra equations shortly.

## Relationship Between Eigenvalues of S and the Equilibrium Abundances

Based on an “off-diagonal” matrix perturbation analysis (ref.^28^) it is possible to show that the eigenvalues λ_i_ of the community matrix **S** = **DA** of RMT systems and the equilibrium abundances ${N}_{i}^{\ast }$, are simply related, namely: ${\lambda }_{{\rm{i}}}\simeq {s}_{ii}=-\,{N}_{i}^{\ast }$ (see Methods and SI1). The approximation holds in the range $\gamma  < 1$. Thus the critical eigenvalue component ${\rm{\Lambda }}({\bf{S}})={{\rm{\max }}}_{i}\,Re({\lambda }_{i})$, can be well approximated by the minimum equilibrium population $-{N}_{min\,}^{\ast }$:

13 $${\rm{\Lambda }}({\bf{S}})\simeq -\,{N}_{min}^{\ast }.$$

The critical eigenvalue component is often used as a stability or resiliency index^5,29,30^. When ${\rm{\Lambda }}({\bf{S}}) < 0$, the system is technically locally stable. However, the smaller or more negative is ${\rm{\Lambda }}({\bf{S}}),$ the more resilient is the community in terms of the time taken to return to equilibrium after a small perturbation. Arnoldi *et al*. (2017)^31^ write that this form of “resilience is the most commonly used stability measure in theoretical ecology” (30). Equation 13 implies that the larger is the biomass of the rarest species $({N}_{min}^{\ast })$, the stronger is the stability or resilience of the system since it will ensure a more negative ${\rm{\Lambda }}({\bf{S}})$, and faster return-time to equilibrium after perturbation^5,29,30^.

Since feasibility requires that the smallest equilibrium population ${N}_{min}^{\ast }$ is positive $({i}{.e}{.},\,{N}_{min}^{\ast } > 0)$, then Eq. 13 makes transparent that in the regime $\gamma  < 1,$ feasibility is linked to both local stability (which requires ${\rm{\Lambda }} < 0$) and resilience. Note this result does not depend on any assumptions about randomness of the perturbation matrix **B**. In the feasible regime, resilience of the most simple or the most complex network, is entirely dependent on the smallest equilibrium abundance, and not directly determined by network properties such as topology, modularity, clustering, and connectedness.

**Figure 4:**
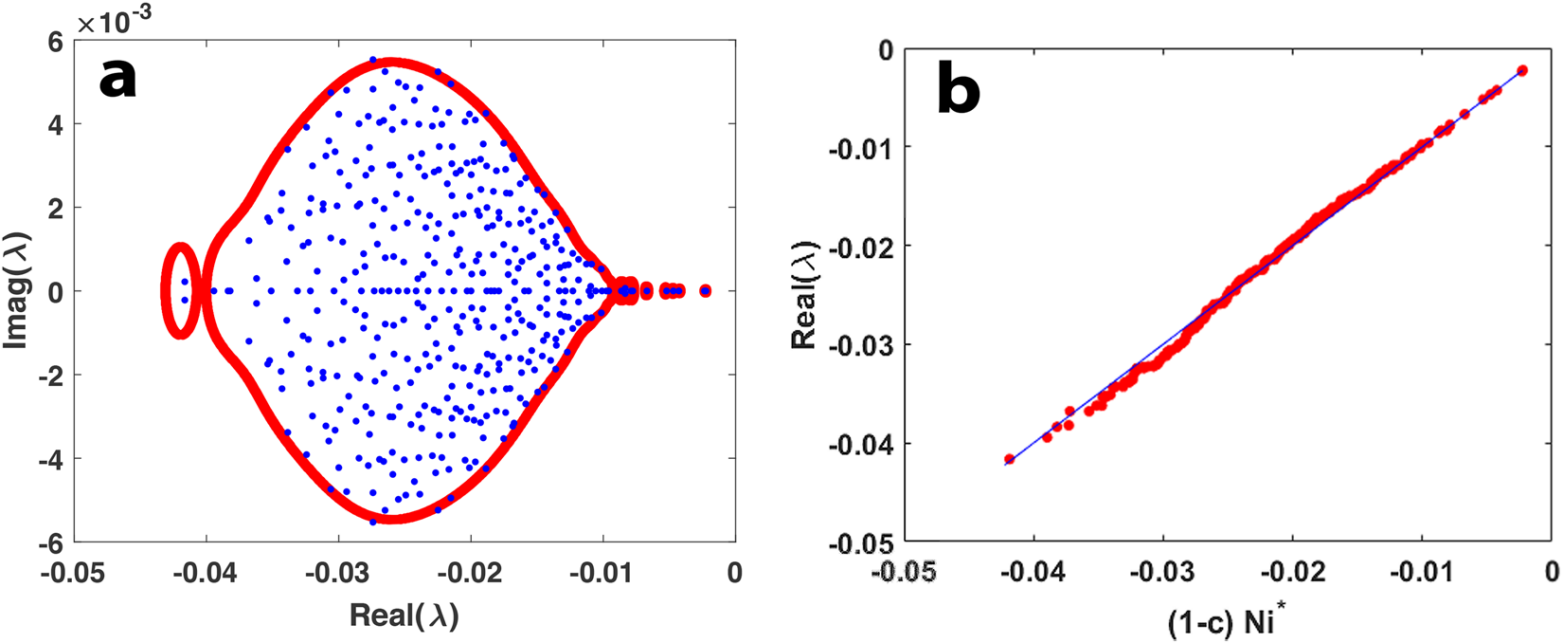
Competition community with *n* = 400, *γ* = *0*.*3*, *c* = *0*.*1*. (**a**) Boundary of the eigenvalue distribution (red) is plotted as predicted by Eq. 16 and the actual numerically calculated eigenvalues are given (blue dots). (**b**) The real parts of the eigenvalues of **S** are plotted against the equilibrium populations (1 − *c*)$\,{N}_{i}^{\ast }\,{{\rm{indicating}}}_{{\rm{i}}}\simeq -\,(1-c){N}_{i}^{\ast }$, and the points sit close to the 45 degree line as predicted in the text. For ease of visualisation the single outlying eigenvalue λ = −21.41 has been removed from the plots (see Methods).

To give an indication of the performance of Eq. 13 as an estimator for Λ, results for DD community matrices **S** = **DA** are examined later in Figs 4b and SI4. Some caveats and limitations concerning this approach are discussed in SI4 and 5.

## Extensions and Ecological Examples

### Example 1. Stability and eigenvalues of Lotka-Volterra competition communities

We now proceed to explore a fully defined density-dependent biological model, rather than just an abstract analysis of an arbitrary community matrix with random equilibria populations. The classical Lotka-Volterra (LV) equations serve this purpose well, being one of the most successful models for studying large complex systems^2–7,25^. For an *n*-species system, the equation for the abundance of species-*i* is:

14 $$\frac{d{N}_{i}}{dt}={N}_{i}({r}_{i}+\,{\sum }_{j=1}^{n}{a}_{ij}\,{N}_{j})\,\,i=1,2,\ldots ,n.$$

The community matrix for this system may be written as the matrix **S** = **DA**, where now the populations in ${\bf{D}}=diag({N}_{i}^{\ast })$ are actual equilibrium solutions of model Eq. 9, found by setting all rates to zero. Following conventional practice, the intrinsic growth rates *r*_*i*_ are all scaled to unity^7,10–14^ (see SI1), and positive reflecting the implicit presence of resources. Following May, it has also become conventional practice to scale the intraspecific competition so that ${a}_{ii}=-\,1.$ Under these conditions, this effectively scales all equilibrium populations to unity ${{\rm{N}}}_{{\rm{i}}}^{\ast }=1$, when there are no interactions between species. While some generality is lost with this scaling, it nevertheless opens the door to the advantageous possibility of analytical calculations.

The simplest competition community is the “uniform model,” where all coefficients are fixed to the same constant ${a}_{ij}=-\,c$, $0 < c < 1,$ and the system is fully connected (*C* = 1). In this parameter range, the equilibrium is always feasible and stable^10,11^. Hence the deterministic uniform model predicts that large competitive communities will satisfy two potentially advantageous features of viable ecosystems, namely feasibility and stability. We will see nevertheless that these seemingly stable and well-organized systems may be highly fragile in the presence of environmental fluctuations.

In the spirit of May^1^ and Roberts^7^, a large ensemble of competitive communities may be specified all of which, on the average, resemble the uniform model with mean interaction strength $E({a}_{ij})=-\,c.$ The interaction matrix **A** is given coefficients of the form ${a}_{ij}=-\,[c+{b}_{ij}\,],$ where the *b*_*ij*_ are mean zero random perturbations with variance Var(*b*_*ij*_) = *σ*^2^. In this model, environmental fluctuations make the interaction strengths vary about the community’s mean strength of competition. Thus two communities may both have the same average interaction strength -c, but the one undergoing stronger perturbation will show a greater variation in its interaction coefficients. Hence the stochastic model associates increasing disturbance with an increase in *σ*^2^.

The interaction matrix can now be written as

15 $${\bf{A}}=-\,(1-c)[{\bf{I}}+{\bf{B}}{\boldsymbol{^{\prime} }}]-c\,e.{e}^{T}$$

where ${e}^{T}=[1,1,1,\ldots ,1,1],$ the term *ce*.*e*^*T*^ is a rank-one perturbation of the scaled May matrix, and the symbol ′ represents a division by (1 − *c*). Stability of the competition system depends in the usual way, on the eigenvalues of the community matrix **S** = **DA**. It is shown in the Methods that for a competition system, all but one of the eigenvalues of the community matrix **S** = **DA** are *identical* to those for a system in which **S**_**m**_ = **DA**_**m**_, where ${{\bf{A}}}_{{\bf{m}}}=-\,(1-c)[{\bf{I}}+{\bf{B}}{\boldsymbol{^{\prime} }}]$. (The one outlier eigenvalue is discussed in the Methods). Thus we can learn a lot about the true competition community matrix **S** from a study of the simpler matrix **S**_**m**_ = **DA**_**m**_. Certainly, if **S**_**m**_ = **DA**_**m**_ is stable so too is **S** = **DA**.

The eigenvalue support for **S**_**m**_ = **DA**_**m**_ may be found by using Eq. 9 after appropriate adjustment for the factor *(1* *−* *c)*. That is, the eigenvalue density of the matrix **S**_**m**_ = **DA**_**m**_ is nonzero in the region of the complex plane, satisfying:

16 $$T={\sum }_{i=1}^{n}\frac{{({N}_{i}^{\ast })}^{2}}{(z^{\prime} +{N}_{i}^{\ast })(\bar{z}^{\prime} +{N}_{i}^{\ast })}\ge {(1-c)}^{2}/{\sigma }^{2}$$

where $$z=z^{\prime} (1-c).$$

Figure 4a plots the eigenvalue distribution of the community matrix **S** for a typical *n* = 400 species competition community (*γ* = 0.*3*, *c* = *0*.*1*) and we see that the red boundary for the support of the eigenvalues predicted by Eq. 16 at equality, is an excellent fit. Note that the eigenvalues in Fig. 4a are all in close vicinity, and are referred to as the “bulk” eigenvalues^23^. There is also an outlying^23^ real eigenvalue λ = −21.41 not shown in the figure as it is completely out of scale. For competition communities, the outlying eigenvalue is an outcome of having added a constant term -*c* to all interaction coefficients ${a}_{ij}=-[c+{b}_{ij}\,]$, or a rank-one perturbation (see Methods).

Hence, using previous arguments and Eq. 16 (setting $z=0$), we can understand that if **A**_**m**_ is stable, both **S**_**m**_ = **DA**_**m**_ and **S** = **DA** are stable, and it may be simply deduced from Eq. 16 that:

17 $${\rm{All}}\,{\rm{feasible}}\,{\rm{competition}}\,{\rm{systems}}\,{\rm{are}}\,{\rm{locally}}\,{\rm{stable}}\,{\rm{if}}\,\gamma =\frac{\sqrt{n}\sigma }{1-c} < 1,$$

apart from rare statistical exceptions (see also refs^10,11^). To remind the reader, the key assumptions behind this result is that parameters are such that the “uniform competition model,” is stable with $0 < c < 1,$ and the system is fully connected (*C* = 1).

In Fig. 4b, the real parts of the eigenvalues of **S** are plotted against the equilibrium populations (1 − c) ${N}_{i}^{\ast }\,{\rm{indicating}}\,{{\rm{\lambda }}}_{{\rm{i}}}\simeq -\,(1-c){N}_{i}^{\ast }$, and the points sit close to the 45 degree line as predicted by the theory, Eq. 13, when adjusted for competition.

It is important to note that Ahmadian *et al*.^16^, in their study of the eigenvalue distribution of **S** = **DA**, assume that the matrix **D** is fixed and deterministic while the matrix **A** is random. However, in our case the matrix **D** = diag(${{\rm{N}}}_{{\rm{i}}}^{\ast }$) is composed of the equilibrium populations which depend on the interaction matrix **A** as it changes from realization to realization. Thus given individual realizations ${\bf{A}},\,\,{\bf{A}}{\boldsymbol{^{\prime\prime} }},\,{\bf{A}}{\boldsymbol{^{\prime\prime} }}$ etc. of sufficiently large random matrices, all of these would approximately exhibit the same eigenvalue distribution. However, the analogous set of random matrices ${\bf{D}}{\bf{A}},\,\,{\bf{D}}{\boldsymbol{^{\prime} }}{\bf{A}}{\boldsymbol{^{\prime} }},\,\,{\bf{D}}{\boldsymbol{^{\prime\prime} }}{\bf{A}}{\boldsymbol{^{\prime\prime} }}$ etc. considered in the current manuscript can potentially have different eigenvalue distributions. However, although differences do occur, in practice they are relatively minor in the parameter range required for feasible systems (i.e., where $\gamma \ll 1$, as will be shortly demonstrated). Moreover, the key result given by Eq. 17 should remain unaffected by this limitation.

### Example 2. Feasibility implies stability in the ensemble LV model

We have just seen that when $\gamma  < 1$ *all feasible systems* of the structure examined here are locally stable (apart from rare statistical exceptions). Without having gained an understanding of the eigenvalue relationship between the interaction matrix **A** and **S** = **DA**, this result would not be available to us. An important question to ask now, is whether feasible RMT competition systems are always locally stable? This would be the case if it could be shown that feasible systems only occur when $\gamma  < 1.$

We therefore need to estimate the parameter regime where feasible systems can be found. This requires determining analytically the conditions all *n* species have positive equilibrium values with ${N}_{i}^{\ast } > 0\,i=1,2,\ldots ,n.$ The mathematical techniques required to accomplish this may be found in ref.^11^ (and in Supplementary Information of ref.^10^, but given here because the approach and result is critical to the main findings in this paper. The probability that a particular system is feasible *Pr*(Feasible) requires first the determination that a typical single species has positive population i.e., $p=Pr({N}_{i}^{\ast } > 0)$, which we proceed to find.

#### Competition communities

Based on the equilibrium condition AN^*^ = **−1** from Eq. 14, when $\gamma  < 1$, a first-order approximation of the equilibrium populations of the competition equations is

18 $${N}_{i}^{\ast }\approx \kappa (1-{\sum }_{j=1}^{n}{b}_{ij}^{\text{'}}\,)$$

where *κ* is a positive constant and the symbol ′ represents a division by (1 − *c*).

We let ${X}_{i}=1-{\sum }_{j=1}^{n}{b^{\prime} }_{ij}$ and note that by the Central Limit Theorem, *X*_*i*_ is a normally distributed random variable with mean and variance: $\langle {X}_{i}\rangle =1$, $Var({X}_{i})\approx {\gamma }^{2}$ where $\gamma =\surd n\sigma /(1-c)$. Thus ${\rm{p}}$ = $Pr({N}_{i}^{\ast } > 0)$ = $Pr({X}_{i} > 0)$ = $Pr(Z < \tfrac{1}{\sqrt{Var({X}_{i})}})$, where *Z* is the standardized normal variate, namely *Z* ~ *N*(0, 1). Thus $p=Pr({N}_{i}^{\ast } > 0)$ is purely a function of the single aggregated parameter *γ* i.e., *p* = *p*(*γ*).

Since the species are relatively independent, and since the *n*-species all have similar characteristics, a first order estimate of system feasibility is given by the probability that *all n*-species equilibria are greater than zero, namely:

19 $$Pr({\rm{Feasible}})=p{(\gamma )}^{n}.$$

**Figure 5:**
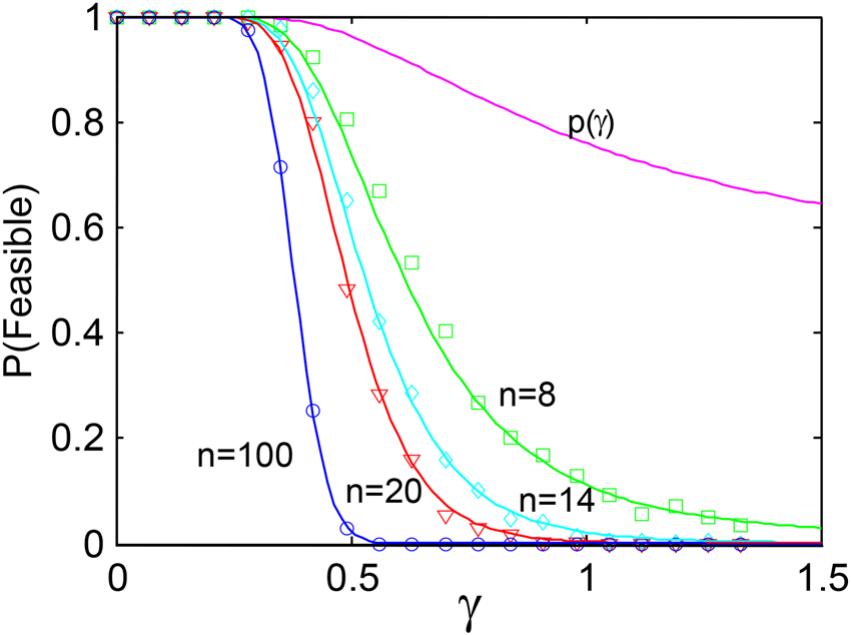
The probability of feasibility, *Pr*(Feasible), as a function of disturbance *γ*, for *n*-species competition with different community sizes *n* = 1, 8, 14, 20, 100. Each probability marked by a square, circle, etc is the proportion of feasible systems in 500 runs of Eq. 14. Coloured lines give analytical predictions from Eq. 19. Figure from Stone^10,11^.

A plot of *p*(γ) is evaluated numerically in Fig. 5 as a function of *γ* and Pr (Feasible) = *p*(γ)^n^ is also plotted as coloured lines for communities of different sizes from *n* = 14 to *n* = 100. Due to the power *n* in Eq. 21, the feasibility fraction can only be substantially positive for *γ* ≪ 1. Thus feasible systems are only found for

20 $$\gamma \ll 1.$$

In addition to the theoretical results, Fig. 5 provides a plot of the percentage of feasible competition Lotka-Volterra models from a simulated random ensemble of systems, as a function of disturbance *γ* The graphs corroborate that the larger the number of species *n*, the more difficult it becomes to generate a feasible system. One also sees that the analytical predictions based on Eq. 19 are accurate since they sit close to the numerical equilibrium analyes (circles) of the Lotka-Volterra systems in Fig. 5.

Return now to our initial query: Are all feasible systems stable? Results for competition communities indicate that we should not expect to find feasible systems unless *γ* ≪ 1, (refer to discussion related to Eq. 20). Yet from Example 1 above, it was found that all feasible systems are stable as long as *γ* < 1. This implies *all feasible competition systems must be stable*. It also explains both in intuitive terms and theoretical terms (details in SI3) why there are no feasible-stable systems when *γ* > 1.

#### Mutualistic communities

For the case of mutualist systems, the result can be generalized further. Consider the LV *n*-species mutualistic system:

21 $$\frac{d{N}_{i}}{dt}={N}_{i}({r}_{i}+\,\sum _{j=1}^{n}{a}_{ij}\,{N}_{j})\,i=1,2,\ldots ,n.$$

in which ${a}_{ij}\ge 0,\,{a}_{ii}=-\,1,$and it is assumed the matrix **A** is strongly connected (i.e., irreducible). The birth rates *r*_*i*_ > 0 and at least one *r*_*i*_ > 0. (This is just Eq. 9 for competition communities, but with signs of interactions made appropriate for mutualistic systems). A simple application of M-matrix theory establishes that all feasible systems are locally stable^11,32^. More recently, this result has been extended and it has been shown that the mutualistic system Eq. (23) possess a globally asymptotically stable feasible equilibrium iff **A** is locally stable^32^. This leads to an interesting situation with regards to mutualist systems, in that local stability of the interaction matrix **A** and feasibility are tied in a manner that ensures that *all feasible mutualistic systems are stable*.

**Figure 6:**
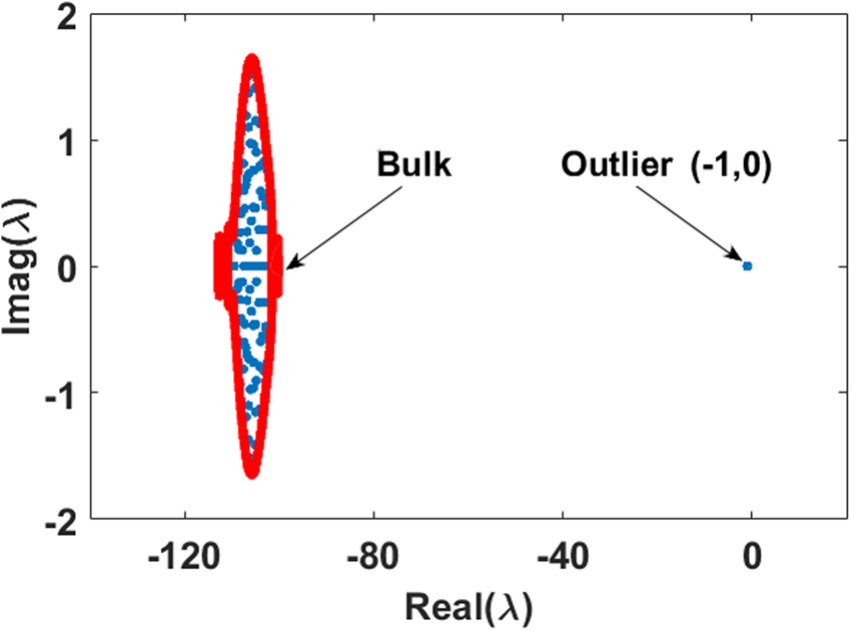
Eigenvalue distribution in the complex plane of community matrix **S** = **DA** for an *n* = 100 species mutualistic community (*m* = 0.01 = −*c*, *σ* = 0.02). Boundary of the eigenvalue distribution (red) is plotted as predicted by Eq. 16 and the actual numerically calculated eigenvalues are given (blue dots). The stability of **S** depends on the critical outlier eigenvalue ${\rm{\Lambda }}\,=-\,1$ located at (−1, 0).

The equilibrium ${N}_{i}^{\ast }$are solutions of the the LV model (eq. 21) whereby AN* = **−1**. Thus the community matrix SN* = −1 N*, has an eigenvalue of −1 (see Methods). This “outlier” eigenvalue is well separated from the “bulk” as shown in Fig. 6. The critical eigenvalue of **S** proves to be ${\rm{\Lambda }}=-\,1$ for all values *m* for which there is a feasible equilibrium. Thus the degree of mutualistic interaction *m* has *no* impact on the resiliency of a feasible equilibrium.

## Discussion

Many previous studies of biological networks have been unable to determine the stability properties of the community matrix **S** = **DA** for large complex random matrix systems. This is considered an unsolved and open problem^6,10^. Here a simple solution is presented based on the trace statistics of random matrices. For feasible RMT systems, it was shown that the community matrix **S** = **DA** transitions from stability to instability, at exactly the same parameter values for which the interaction matrix **A** transitions. Thus for a large feasible system with **D** > 0, stability of the interaction matrix **A** implies stability of the community matrix **S** = **DA**. The theoretical prediction depends on the assumption that the matrices **D** and **A** are independent, but the presence of correlations appears only minor in the present context. Simulations indicate the dependence is weak and appears to have little impact (see SI6).

Feasible RMT systems were shown to be nearly always stable in the regime γ < 1. However, for the classical ecological RMT models examined here, feasible systems are rarely found when γ > 1. For this reason all feasible systems examined were stable. While these results may in some sense be model dependent, they should provide a good general characterization of how the addition of heterogeneity and external perturbations will affect any feasible stable system. Namely, as heterogeneity and disturbance increases, the feasibility of the system will be particularly sensitive to the heterogeneity in interaction, and feasibility will be lost often even before the transition from stability to instability of the interaction matrix. Future research in network science may benefit from shifting focus to study those factors which promote system feasibility^6,10,15^.

Finally, if the LV systems are a good guide to real world ecological systems, they inform us that large complex systems may be far more fragile than May’s main result predicts. The models studied here suggest that feasible stable mutualistic, and competition systems can only be found if $\gamma \ll 1\,$(refer to Eq. 20). The same is true for predator-prey systems modeled by setting *c* = *0*. Thus the models indicate the difficulty of assembling large complex ecosystems that are feasible, and in addition indicate their fragility to perturbation in interaction strength. However, those RMT systems that can be assembled and that are feasible, are nearly always found to be automatically stable. This suggests the possibility that many large ecological networks *observed* in the real world (i.e., feasible systems) may be endowed with some underlying intrinsic stability, a possibility that needs further investigation.

## Methods

### Boundary of eigenvalue distributions

Ahmadian *et al*.^16^ studied the eigenvalue distribution of matrices having forms similar to May’s neutral interaction model, ${\bf{S}}={\bf{D}}{\bf{A}}=-\,{\bf{D}}+{\bf{D}}{\bf{B}}$, where **B** is a random matrix.and **D** =  *−diag(*${N}_{i}^{\ast }$*)* a deterministic matrix. Ahmadian *et al*.^16^ demonstrated that for large *n*, the eigenvalue density of the matrix **S** is nonzero in the region of the complex plane, satisfying:

22 $$\,trace[{({{\bf{M}}}_{z}{{\bf{M}}}_{z}^{\dagger })}^{-1}]\ge 1/{\sigma }^{2}\,{\rm{where}}\,{{\bf{M}}}_{{\boldsymbol{z}}}={{\bf{D}}}^{-1}(\,\,z{\bf{I}}-{\bf{D}}),$$

where the complex variable *z* = *x* + *iy*, and the strength of random perturbations ${\rm{Var}}({{\rm{b}}}_{{\rm{ij}}})={{\rm{\sigma }}}^{2}$. The notation ${{\bf{M}}}_{z}^{\dagger }$ indicates the complex conjugate of the transposed matrix of **M**_z_.

With interaction connectance operating, then with probability *C* the updated entries of **B** are ${b}_{ij}^{u}={{\rm{b}}}_{{\rm{ij}}}$, and probability (1 − *C*) the updated entries are $\,{b}_{ij}^{u}=0.$ Being a product of random variables, ${\rm{Var}}({b}_{ij}^{u})=C{{\rm{\sigma }}}^{2}$, and the term on the RHS of the inequality of Eq. 8 should thus be replaced by 1/(*Cσ*^2^). Hence, with connectance *C*, the eigenvalue density of the community matrix is nonzero in the region of the complex plane, satisfying:

23 $$trace[{\bf{Q}}]\ge 1/(C{\sigma }^{2}),$$

where the matrix ${\bf{Q}}={(\bar{z}{\bf{I}}+{\bf{D}})}^{-1}{\bf{D}}{(z{\bf{I}}+{\bf{D}})}^{-1}$, **D** is diagonal, and the *i*’th diagonal entry is of the form ${({N}_{i}^{\ast })}^{2}/[(z+{N}_{i}^{\ast })(\bar{z}+{N}_{i}^{\ast })]$.

Thus the region for which the eigenvalue density of the matrix **S** is nonzero corresponds to the region in the z-plane where:

24 $$T={\sum }_{i=1}^{n}\frac{{({N}_{i}^{\ast })}^{2}}{(z+{N}_{i}^{\ast })(\bar{z}+{N}_{i}^{\ast })}\ge 1/[C{\sigma }^{2}].$$

### The Lotka-Volterra competition model (*c* > 0)

(or mutualist when *c* < 0), has an interaction matrix of the form:

25 $${\bf{A}}=(1-{\rm{c}})[-{\bf{I}}+{{\bf{B}}}^{\text{'}}]-{\rm{c}}\,{\rm{e}}.{{\rm{e}}}^{{\rm{T}}}={{\bf{A}}}_{{\bf{m}}}-{\rm{c}}\,{\rm{e}}.{{\rm{e}}}^{{\rm{T}}}$$

where ${{\bf{A}}}_{{\bf{m}}}=(1-{\rm{c}})[-{\bf{I}}+{{\bf{B}}}^{\text{'}}]\,{\rm{and}}\,{{\rm{e}}}^{{\rm{T}}}=[1,\,1,\,1,\ldots ,\,1,\,1]$. Tao^23^ has shown that for large systems, e is a good approximation to the right eigenvector of **A**_**m**_. Thus based on rank-one perturbation theory, if **A**_**m**_ has eigenvalues ${{\rm{\lambda }}}_{1},\,{{\rm{\lambda }}}_{2},\mathrm{.}.,{{\rm{\lambda }}}_{{\rm{n}}-1},\,{{\rm{\lambda }}}_{{\rm{n}}},$then all but one of the eigenvalues of **A** may be approximated by the eigenvalues of **A**_**m**_, and are approximately λ_1_ − *nc*, ${{\rm{\lambda }}}_{2},\,\mathrm{.}.,\,{{\rm{\lambda }}}_{{\rm{n}}-1},\,{{\rm{\lambda }}}_{{\rm{n}}}$ (see Methods). Of course, the circular law holds^23–25^, and the eigenvalues of **A**_**m**_ are distributed in a circle of radius $\gamma =\sqrt{n}\sigma$ and centred on the x-axis at the point (−(1 − *c*), 0).

For feasible Lotka-Volterra systems with birth-rates scaled so that r_1_ = 1, then the community matrix **S** = **DA** always has the real eigenvalue λ_1_ = −1 and the associated real eigenvector N* = **De** (see Stone^10,11^). All but one of the eigenvalues of the community matrix **S** = **DA** are exactly identical to the eigenvalues of the matrix **DA**_**m**_, whether or not **A** is a random matrix. Thus if **S** = **DA** has eigenvalues ${{\rm{\lambda }}}_{1}=-\,1,\,{{\rm{\lambda }}}_{2},\mathrm{.}.,\,{{\rm{\lambda }}}_{{\rm{n}}-1},\,{{\rm{\lambda }}}_{{\rm{n}}},$ then the eigenvalues of **S**_**m**_ = **DA**_**m**_ are exactly given by ${{\rm{\lambda }}}_{1}=(-1-{\rm{c}}{\sum }^{})$, ${{\rm{\lambda }}}_{2},\mathrm{.}.,{{\rm{\lambda }}}_{{\rm{n}}-1},\,{{\rm{\lambda }}}_{{\rm{n}}}.$ Here ${\rm{\Sigma }}={{\rm{\Sigma }}N}_{i}^{\ast }$ This property was noted by Brauer^33^ and found independently by Ding and Zhou^34^, Langville & Meyer (2004)^35^ and Stone (1988), and referred to as the “Google-matrix” property in ref.^10^.

Since **A**_**m**_ = (1 − c) [−**I** + **B**′], its eigenvalue support may be found by using Eq. 5 after appropriate adjustment for the factor (*1* − *c*). That is, the eigenvalue density of the matrix **S**_**m**_ = **DA**_**m**_ is nonzero in the region of the complex plane, satisfying:

26 $$T={\sum }_{i=1}^{n\,}\frac{{({N}_{i}^{\ast })}^{2}}{({z}^{\text{'}}+{N}_{i}^{\ast })(\,{\bar{z}}^{\text{'}}+{N}_{i}^{\ast })}\ge {(1-c)}^{2}/{\sigma }^{2}$$

where *z* = *z*′(1 − *c*). Note that these systems are assumed to be fully connected with *C* = 1 (although see SI5 for the case *C* < 1). In addition, Lotka-Volterra systems, the community matrix **S** always has the eigenvalue *λ*_1_ = −1 which may appear as an **outlying eigenvalue**^23^ (see eg., Fig. 5).

### Relation between population equilibria ${N}_{i}^{\ast }$and eigenvalues ${\lambda }_{i}$

**Figure 7:**
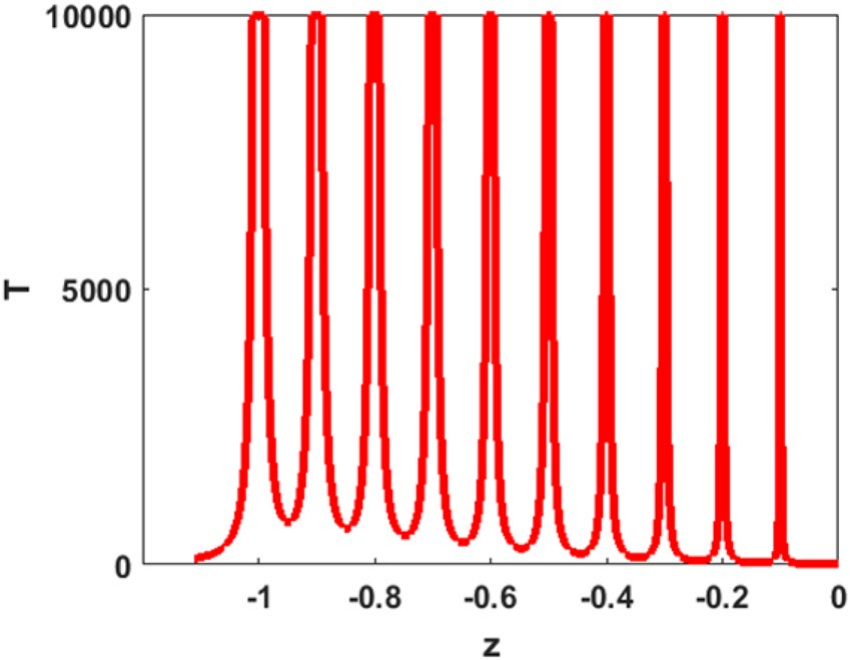
Plot of T in LHS of Eq. 9 as a function of *x* = −*z*, which is real, for an *n* = 10 species community with ${N}_{1}^{\ast }=0.1,\,{N}_{2}^{\ast }=0.2,\,{N}_{3}^{\ast }=0.3,\ldots \ldots ,\,{N}_{10}^{\ast }=1$. Singularities occur when $x=-\,{N}_{i}^{\ast }$.

Returning to inequality Eq. 9, note that the left-hand-side of the expression for *T* has a singularity for those values of *z* for which $z=-\,{N}_{i}^{\ast }.$This is visualised in Fig. 7 where *T* is plotted as a function of *z* for a hypothetical *n* = 10 species community with ${N}_{1}^{\ast }=0.1,\,\,{N}_{2}^{\ast }=0.2,\,\,{N}_{3}^{\ast }=0.3,\ldots \ldots ,\,\,{N}_{10}^{\ast }=1.$ For purposes of illustration, it is assumed that *z* is a real number in the interval [0, 1]. The function *T* clearly explodes at all points where $z=-\,{{\rm{N}}}_{{\rm{i}}}^{\ast }$. In this cut in the complex plane, the eigenvalues are predicted to be located on the x-axis (real-axis) at those points where $T > 1/[C{\sigma }^{2}]$ = 1000 (in this example). It is clear that the eigenvalues must lie close to the population equilibria ${\lambda }_{i}\simeq -\,{N}_{i}^{\ast }$. In general, the smaller the population −${N}_{i}^{\ast }$, the more exacting is the approximation as can be seen from comparing the slopes of the graphs about the equilibria (and as can be verified by examining *∂T*/*∂x*).

### The eigenvalue approximation

$$\,{\lambda }_{i}(S)=-\,{N}_{i}^{\ast }+O(\parallel DB{\parallel }^{2})$$

For the basic “neutral interaction” model, consider A = −**I** + **B**, where **I** is the identity matrix and ${\bf{B}}$ a matrix of perturbations that are not necessarily random. In the extreme limiting case, when all off-diagonal interspecific interactions are set to zero (γ = 0), then **A** = −**I** and the community matrix is simply ${\bf{S}}=-\,{\bf{D}}=-\,diag({N}_{i}^{\ast })$, and the eigenvalues ${{\rm{\lambda }}}_{{\rm{i}}}({\bf{S}})={{\rm{\lambda }}}_{{\rm{i}}}({\bf{D}}{\bf{A}})=-\,{N}_{i}^{\ast }.$

When interspecific interactions are switched on (γ > 0), and for reasonable assumptions (see also SI4 and ref.^28^), the “off-diagonal” perturbation expansion is:

27 $${{\rm{\lambda }}}_{{\rm{i}}}({\bf{D}}{\bf{A}})={{\rm{\lambda }}}_{{\rm{i}}}({\bf{D}}[\,-\,{\bf{I}}+{\bf{B}}])={{\rm{\lambda }}}_{{\rm{i}}}(-{\bf{D}})+{{\rm{v}}}_{{\rm{i}}}^{{\rm{T}}}{\bf{D}}{\bf{B}}{{\rm{v}}}_{{\rm{i}}}+O(\parallel {\bf{D}}{\bf{B}}{\parallel }^{2}).$$

Here v_i_ is a normalised eigenvector of **D** such that **D**v_i_ = λ_i_(**D**)v_i_ (The spectral norm **E** = σ_max_ (**E**) in terms of singular values may be used). The success of the approximation ${\lambda }_{i}\simeq -\,{N}_{i\,}^{\ast }$, is because the first-order perturbation term vanishes (${{\rm{v}}}_{{\rm{i}}}^{{\rm{T}}}{\bf{D}}{\bf{B}}{{\rm{v}}}_{{\rm{i}}}=0)$, and

28 $${{\rm{\lambda }}}_{{\rm{i}}}({\bf{D}}{\bf{A}})={{\rm{\lambda }}}_{{\rm{i}}}(-{\bf{D}})+{\rm{O}}(\parallel {\bf{D}}{\bf{B}}{\parallel }^{2})\simeq -\,{N}_{i}^{\ast },$$

leaving a small quadratic error term. (More details are given in SI4).

The intuition behind approximation Eq. 13 may be understood as follows. In the extreme limiting case, when all off-diagonal interspecific interactions are set to zero (γ = 0), the eigenvalues of **S = DA** have precisely the same magnitude as the equilibrium population values with ${\lambda }_{i}=-\,{N}_{i}^{\ast }$, and therefore this holds exactly. Figure 2c shows a situation very near to this case with γ = 0.01, for which there are many weak off-diagonal interspecific interactions, and nearly all of the eigenvalues sit on the real axis in close proximity to the equilibrium population values ${\lambda }_{i}\simeq -\,{N}_{i}^{\ast }$. Denoting the smallest and largest equilibrium population as ${N}_{min}^{\ast }$ and ${N}_{max}^{\ast }$ (in blue), then all eigenvalues should be wedged in the interval −[${N}_{max}^{\ast },\,{N}_{min}^{\ast }$] in the complex plane as seen in Fig. 2c between the two demarked points in blue. But this holds to a good approximation even when the intensity of the perturbed interactions is increased, as shown for γ = 0.2 in Fig. 2b. See SI4 for more examples and a discussion of caveats.

### Data Availability Statement

No datasets were generated or analysed during the current study.

### Note

After completion and submission of this manuscript (first submitted June 2017), it was found that some results overlap with an unpublished manuscript of Gibbs, Grilli, Rogers, Allesina found on BioArxiv 1708.08837v1 (submitted 29/8/17), but were obtained by different methods.

## Electronic supplementary material

Supplementary Information
