# Supplementary material for: The feasibility and stability of large complex biological networks: a random matrix approach
Source: Sci Rep. 2018 May 29;8:8246. doi: 10.1038/s41598-018-26486-2 (PMC5974107; doi:10.1038/s41598-018-26486-2)
Supplement: Supplementary file 1 — Supplementary Information [file 41598_2018_26486_MOESM1_ESM.docx]

**SUPPLEMENTARY INFORMATION**

**Title: “The feasibility and stability of large complex biological networks: a random matrix approach“ by** Lewi Stone

**SI1 Lotka-Volterra Ensemble Model and Competition**  **2**

**SI2 Stability of A implies stability of S=DA, for feasible systems 3**

**SI3 Feasibility of competition systems is lost before 4**

**stability of the interaction matrix A.**

**SI4 The eigenvalue approximation:** $\lambda_{i}\left( \mathbf{S} \right)=-N_{i}^{*}+O(\left\| DB \right\|^{2})$. **5**

**Limitations when interaction perturbations (γ) are large**

**SI5 Resiliency of competition versus mutualist communities: 10**

**A comparison of methods**

**SI6: The effects of correlations between D and S=DA 11**

**Additional References 14**

**SI1 Lotka-Volterra Ensemble Model and Competition:**

**Parameters:** Let *N_i_* be the abundance of the i’th species, the Lotka-Volterra equations for a total of *n*-species can be written in the usual form:

$\frac{dN_{i}}{dt}= N_{i} (r_{i}+\sum_{j} a_{ij} N_{j})$ *i=1,2,..., n* SI1.1

Here *r_i_* is the birth-rate of the *i*’th species, and $a_{ij}$the interaction coefficient representing the effect species-*j* has on species*-i*. The equations have been normalized as recommended by taking all species to be self-regulated with *a_ii_*= -1. This follows some of the more popular scalings that have appeared in the literature (see eg., Gilpin and Case (1976, 1981), Pomerantz and Gilpin (1979); Roberts (1974, 1984, 1989); Kokkoris & Janssen (2002); Janssen & Kokkoris (2003), Stone & Roberts (1991); Roberts & Stone (2004)), and Rohr et al. (2015)).

***The Ensemble Competition Model:*** As in the main text, interspecific interactions are taken to be of the form

$a_{ij}=-\left( c+b_{ij} \right)<0 .$

Here the $b_{ij}$ are random perturbations having mean zero and variance Var(*b_ij_*)=$\sigma^{2}$. The *b_ij_* are selected randomly so that $<b_{ij}> =0$and $Var\left( b_{ij} \right)=\sigma^{2}$. It is often practical to select the *b_ij_* randomly (uniformly) from the interval $\left[ -cv, +cv \right]$with spread $0\leq v\leq1$, so that $<b_{ij}> =0$and $Var\left( b_{ij} \right)=\frac{c^{2}v^{2}}{3}=\sigma^{2}$.

Thus pairwise interactions have mean strength term <*a_ij_*>=–c and variance Var(*b_ij_*)=${Var(a_{ij})=\sigma}^{2}$ and all$a_{ij}<0$. By setting $b_{ii} =0$, we follow Allesina and Tang (2015) who write: “For simplicity we may set the diagonal entries of **B** to be identically zero, and the eigenvalue does not deviate appreciably from the circular law, when *n* is sufficiently large.”

Competing species are generally modeled with positive intrinsic growth rates *r_i_,* indicating the implicit presence of resources for which to compete. Although some information is lost, for analytical tractability it has become common practice to scale all the growth rate parameters *r_i_* = 1 (Gilpin and Case (1976, 1981), Pomerantz and Gilpin (1979); Roberts (1974, 1984, 1989); Kokkoris & Janssen (2002); Janssen & Kokkoris (2003), Stone & Roberts (1991); Roberts & Stone (2004)),). This in effect scales to unity each equilibrium population in the absence of other species. Thus the competition system has equilibrium populations which are solutions of the equation **AN^*^=-1.**

The community matrix is given b*y* **S=DA** where **D***=diag(Ni*)* and **A***=(a_ij_)* is the matrix of interactions. Note that **D**>0 for a feasible system with all positive equilibrium populations. Also noteworthy, is the relationship $\mathbf{SN}^{\mathbf{*}}\mathbf{=D}\mathbf{AN}^{\mathbf{*}}\mathbf{=-D.1=-}\mathbf{N}^{\mathbf{*}}$ Thus the vector $\mathbf{N}^{\mathbf{*}}$is a right eigenvector of **S=DA**, and λ = -1 is always an eigenvalue.

**SI2 Stability of A implies stability of S=DA, for feasible systems (D>0)**

It is argued here that for large feasible systems (**D**>0), the stability of **A** implies stability of **S=DA**, not just in a statistical sense, but for individual matrices **A** and **S**  (see eqn.12 in main text). This property (which has close similarities to “D-stability” (Johnson 1974)) is certainly not a general property for all matrices, and there are many known counterexamples. An important ingredient of the argument here comes from our knowledge of the spatial distribution of the eigenvalues of both **A** and **S** in the complex plane, as described in the main text (eqns.9-11).

Recall that the eigenvalues of the matrix **A** fall in a circle in the complex plane with radius $\gamma$ as in Fig.1a. There we see that for small $\gamma<1$ the eigenvalues sit in the LHS of the plane. Consider now the properties of each new matrix **A** for each value of $\boldsymbol{\gamma,}$ as$\boldsymbol{\gamma}$ is increased incrementally from zero. In particular, as$\gamma$ increases from zero, the RHS of the circular distribution will eventually touch the y-axis. The point of contact occurs at the origin (see Fig.1a). This is in fact the point of instability for **A**, since it is characterized by the real eigenvalue $\Lambda$ = $\max_{i} {Re(}_{i}(\mathbf{A}))=0$ (for large *n*) and hence at this point the determinant formula ensures $\left| \mathbf{A} \right|=\prod_{j} \lambda_{j}(\mathbf{A})=0$. Similarly, the determinant formula for **S** can be written as

$\left| \mathbf{S} \right|$= $\prod_{i=1}^{n} \lambda_{i}(\mathbf{S})=\left| \mathbf{DA} \right|=\left| \mathbf{D} \right|\left| \mathbf{A} \right|=\prod_{1}^{n} N_{i}^{*}\left| \mathbf{A} \right|=\prod_{1}^{n} N_{i}^{*}\prod_{i=1}^{n} \lambda_{i}(\mathbf{A})=0$.

In short, for large feasible systems ($N_{i}^{*}>0),$ both **S** and **A** both share a zero eigenvalue at this point.

We see that when $\gamma$ increases from zero, and the first eigenvalue of **A** passes through the origin and enters the RHS of the complex plane, then the *first* eigenvalue of **S** must also enter the RHS of the complex plane. Hence the eigenvalues of **S** will zero and proceed to enter the RHS of the complex plane exactly when the first eigenvalue of **A** zeroes. This of course assumes the system remains feasible. Thus for large feasible systems $(\mathbf{D}>0)$, we find that

stability of the interaction matrix **A** implies

stability of the community matrix **S=DA**,

where **A** is a random matrix as defined by May *(1).* This is referenced as eqn.12 in the main text.

**SI3 Feasibility of competition systems is lost before stability of the interaction matrix A.**

**a)_For LV-systems there is population blow up as** $\left| \mathbf{A} \right|\boldsymbol{\to}\boldsymbol{0.}$

The following section (a) revises and corrects notes in Supplementary Information of Stone (2016). The approach used here assumes we begin with a feasible stable competition-system, and we then examine what happens as$\boldsymbol{\gamma}$ is increased incrementally from zero. The properties of each new matrix **A** are studied for each value of $\boldsymbol{\gamma,}$ as$\boldsymbol{\gamma}$ is increased incrementally from zero.

It is shown here that competition systems loses feasibility before the interaction matrix **A** becomes unstable. To see this we examine what happens when$\boldsymbol{\gamma}$ reaches the transition from stability to instability and an eigenvalue of interaction matrix **A** zeroes, as indicated in SI2. At this point we know that the interaction matrix determinant $\left| \mathbf{A} \right|=0.$

Consider now the equilibrium equations of the LV model **AN***=**1**, when $\left| \mathbf{A} \right|\to0.$ Let the vectors *R* and $R^{'}$ be two different rows of the interaction matrix **A**, and $R_{i}$ correspond to the *i*’th component in the vector *R*. When solving for equilibrium, after scaling we have an equation of the form **A***x=r*. Without loss of generality, two rows of this equation would read:

$$\sum R_{i} x_{i}=1$$

$$\sum R_{i}^{'}x_{i}=0$$

Hence $\sum(R_{i}-R_{i}^{'})x_{i}=1$ .

A zero determinant $\left| \mathbf{A} \right|=0,$ is equivalent to having two identical rows in **A,** say vectors *R* and $R^{'}$ .

Now$1= \parallel\left( R - R^{'} \right)x\parallel\leq\parallel R - R^{'}\parallel\parallel x \parallel$, and let $\varepsilon=\parallel R - R^{'}\parallel.$

$Then as \varepsilon\to0,$ this implies $\parallel x \parallel\geq\frac{1}{\varepsilon}\to\infty$.

Hence as $\left| A \right|\to0,$ at least one equilibrium population must blow up, say $N_{1}^{*}\to\infty$ . For the competition system (eqn.1) at equilibrium, the first population equation reads:

$-N_{1}^{*} - \sum_{j=1}^{n} a_{1j}N_{j}^{*}$=1 where $a_{\mathrm{ij}}>0.$

The equations ensure that if population $N_{1}^{*}\to+ \infty$, then this must be counterbalanced by another population, say$N_{2}^{*}\to- \infty$. This implies the system must have lost feasibility ($N_{2}^{*}=0)$ at least by the point before when $\left| \mathbf{A} \right|=0$ i.e., before the point $\gamma$ increases to a value$\gamma=1$.

**b)_No feasible systems for** $\boldsymbol{\gamma>1.}$ As before, the properties of each new matrix **A** are studied for each value of $\boldsymbol{\gamma,}$ as$\boldsymbol{\gamma}$ is increased incrementally from zero. We have just seen that feasibility must be lost before the point $\gamma=1$. Beyond $\gamma=1$, we find very few if any feasible systems for large *n* (*n*$\gtrsim$20). This is backed by numerical simulations (eg., see Fig.5), and our analytical findings in the main text, but as yet no rigorous analytical proof is possible. However, in the strong interaction (large$\gamma)$ regime, the argument of Manton (1979) applies. Referring again to the equilibrium equations

$-N_{i}^{*} - \sum_{j=1}^{n} a_{\mathrm{ij}}N_{j}^{*}$=1

and suppose that the $a_{ij}$ are relatively large and equally positive or negative (though even this assumption can be relaxed). Then it would appear the population equilibria $N_{i}^{*}$ are of arbitrary sign, so that the probability $Pr\left( N_{i}^{*}>0 \right)\simeq0.5,$ and the probability of feasibility (that all *n* populations are positive) is $\Pr\left( Feas \right)=(0.{5)}^{n}\to0,$ for large *n*. So there is little chance of finding a feasible system as $\gamma$ is increased beyond $\gamma=1$.

**SI4. The eigenvalue approximation:** $\lambda_{i}\left( \mathbf{S} \right)=-N_{i}^{*}+O(\left\| \mathbf{DB} \right\|^{2})$

**The key principal:** We seek the eigenvalues of the community matrix **S**=**D**+**E**, which should be viewed as an off-diagonal perturbation of **D** by some matrix **E** (that has small magnitude norm). Thus **E** is considered a matrix of small perturbations whose elements are generally much smaller in absolute magnitude than the diagonal elements of **D**, and for which $E_{ii}=0.$ For simple eigenvalues, the first-order perturbation expansion is (Nakatsukasa 2017; Benaych-Georges et al. ; Allez et al. 2014; Shmueli et al. 2012):

$\lambda_{i}\left( \mathbf{D}+\mathbf{E} \right)=\lambda_{i}\left( \mathbf{D} \right)+v_{i}^{T}\mathbf{E}v_{i}+O(\left\| \mathbf{E} \right\|^{2})$ . SI4.1

Here $v_{i}$ is an eigenvector of **D** such that $\mathbf{D}v_{i}=\lambda_{i}\left( \mathbf{D} \right)v_{i}$, and normalised so that $v_{i}^{T}v_{i}=1.$The spectral norm $\left\| \mathbf{E} \right\|=\sigma_{max}\left( \mathbf{E} \right)$may be used, and refers to the largest singular value of the matrix **E**.

In our case, **D** is a diagonal matrix, with all diagonal terms positive $d_{ii}{=N}_{i}^{*}>0,$ due to feasibility, but all other elements zero. The eigenvalues of a diagonal matrix **D** are $\lambda_{i}\left( \mathbf{D} \right)=d_{ii},$ and the eigenvectors *v* are trivially the standard basis vectors E={$\{e_{1},e_{2},{\ldots.., e}_{n}\}$ where $e_{j}$ is the vector with 1 in the j-the position and zeros elsewhere. This has implication that the first-order term in the above approximation eqn.SI4.1 reduces to zero: i.e., $v_{j}^{T}\mathbf{E}v_{j}=e_{j}\mathbf{E}e_{j}=E_{jj}=0$.

Thus the expression eqn.SI4.1 simplifies to: $\lambda_{i}\left( \mathbf{D+E} \right)=\lambda_{i}\left( \mathbf{D} \right)+O(\left\| \mathbf{E} \right\|^{2})$.

Thus the above eigenvalue bound scales quadratically with the perturbation$\left\| E \right\|$, which for small perturbations should be small. In the context of the general “neutral interaction” community matrix, $d_{ii}=N_{i}^{*}$ and $E=DB$ with elements $e_{ij}=N_{i}^{*}b_{ij}$.

$${\lambda_{i}\left( \mathbf{S} \right)=\lambda}_{i}\left( \mathbf{D+DB} \right)=-N_{i}^{*}+O(\left\| \mathbf{DB} \right\|^{2})$$

These same off-diagonal perturbations have recently become of interest in the works of Nakatsukasa (2017) and Allez et al. (2014). The small second-order error term (*O*($\left\| \mathbf{DB} \right\|^{2}$) is often negligible especially in the regime of feasibility.

To give an indication of the performance and robustness of the estimator for the critical eigenvalue component $\Lambda$ (eqn.13), the results for arbitrary DD community matrices **S**=**DA** are given in Fig.S1 below. The random matrices **A** having “neutral interactions” are constructed as described above. The diagonal matrices **D** have diagonal terms $d_{ii}={-N}_{i}^{*}$that are random numbers chosen uniformly in the interval (0,1). Fig.S1 plots both the smallest equilibrium population $N_{min}^{*}$ (red) against the critical eigenvalue component $-\Lambda$ (blue +), calculated numerically as a function of increasing disturbance γ in (a), and against the number of species *n* in (b). The figures indicate a surprisingly accurate match between$-\Lambda$ and $N_{min}^{*},$ as predicted by eqn.8 with almost negligible error. Figs.S1b&d show that the prediction error is almost zero when $\gamma\lesssim1$ (generally <0.1%, as a percentage of $N_{max}^{*}=1$). Significant errors begin to appear when disturbance levels γ are of the same magnitude as $N_{max}^{*}=1$ i.e., when $\gamma\gtrsim1$.


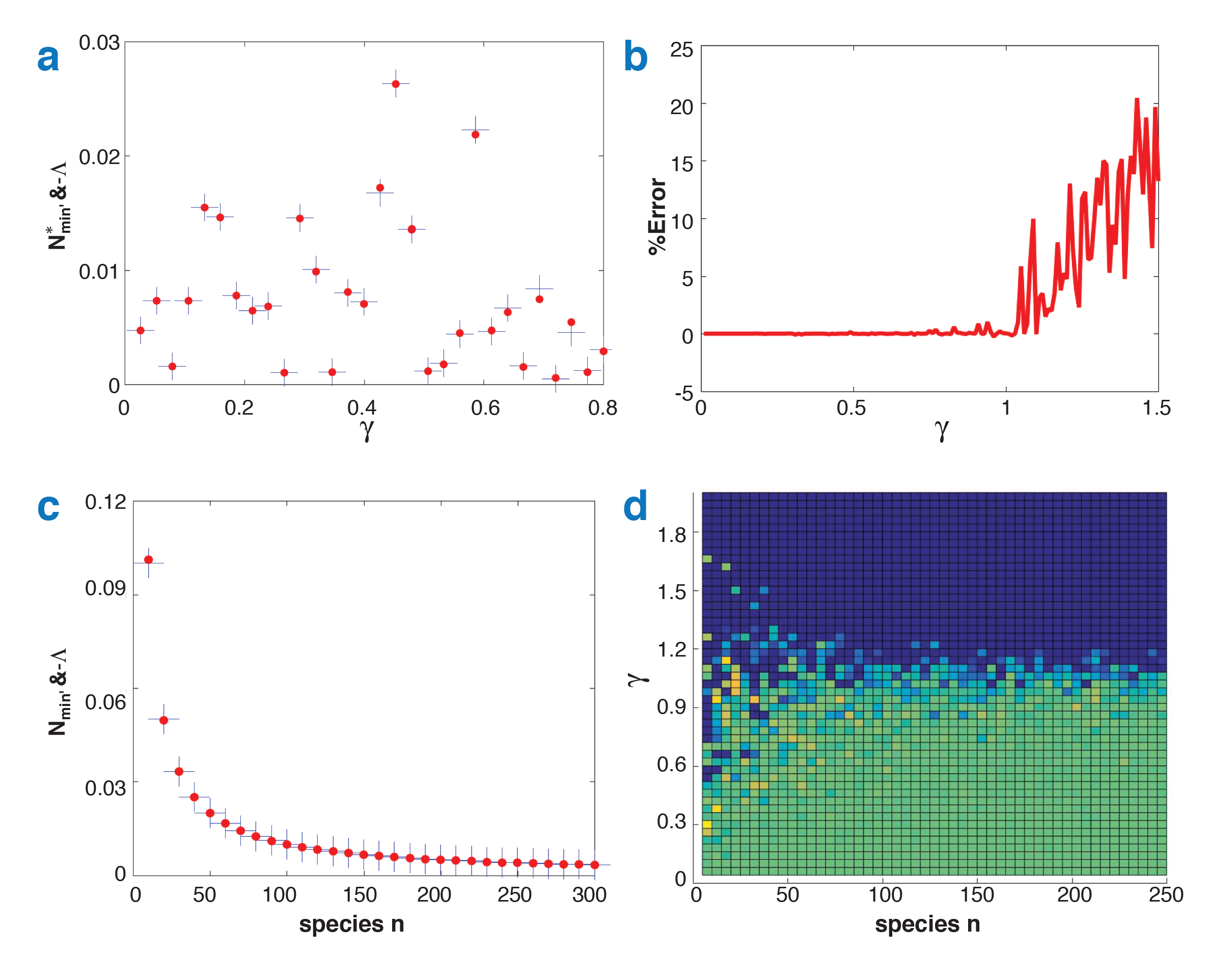
**Figure S1** Neutral-interaction DD community matrix, and the key relationship (eqn.13) between the critical eigenvalue$\Lambda$ and the minimum population equilibrium $N_{min}^{*}$. Populations $N_{i}^{*}$ are chosen randomly in the interval (0,1) and inserted in community matrix **S**=**DA** (for *n*=100). Plots of both $N_{min}^{*}$(blue +) and$-\Lambda$ (red) are graphed a function of γ and show that$\Lambda\simeq{-N}_{min}^{*}$. 30 different random matrices are analysed for 30 values of γ. b) The prediction error E=([-$\Lambda]{-N}_{min}^{*})*100$% (absolute error, as a percentage of $N_{max}^{*}=1)$ plotted as a function of γ. c) Similar to a) but now variables are plotted as a function of number of species *n*$.$ d) Similar to b) but error E is plotted as a function of γ and *n.* Green area indicates error E<0.01%.

Similar results are found for competition communities where the eigenvalue of **S** having maximum real part is given by:

$\Lambda$ = $\max_{i} {Re(}_{i})\simeq{-(1-c)N}_{min}^{*}$.

which readjust eqn.13 for competition.

**Figure S2** LV-competition model with *n*=50 *c*=0.1, connectance C=1, with variables $(1-c)N_{\min}^{*}$and$-\Lambda$ plotted versus disturbance γ. RH panel displays absolute prediction error.

**Limitations when interaction perturbations (γ) are large**

The approximation${s \lambda}_{i}\left( \mathbf{DA} \right)=\lambda_{i}\left( -\mathbf{D} \right)+O\left( \left\| \mathbf{DB} \right\|^{2} \right){\simeq-N}_{i}^{*};$ $\Lambda\simeq{-N}_{min}^{*}$(eqn.13 main text): The populations $N_{i}^{*}$need to be separated out to a reasonable degree and for the case of uniformly distributed population abundances, the relative spread in populations is required to be larger than the relative level of disturbances $\boldsymbol{\gamma}$ i.e., $spread=$ ${(N}_{max}^{*}{-N}_{min}^{*})/N_{max}^{*}\boldsymbol{>\gamma}$. This ensures that the eigenvalues of **D** are well enough separated, so that the addition of the perturbation matrix **DB** ($\mathbf{S=D+DB})$ will not result in approaching singularities or non-simple eigenvalues in **S**, as would be a potential possibility (Allez et al. 2014, Benaych-Georges 2017).

Without loss of generality, the equilibrium populations can be normalised so that $N_{max}^{*}=1$, and our focus requires analysis over the parameter range $0\leq\boldsymbol{\gamma<1}$**.** In the interests of accuracy of eqn.13, to ensure *spread* $\boldsymbol{>\gamma}$***,*** we often choose $N_{i}^{*}$uniformly random from the interval $\left[ 0,1 \right].$ This has the restricting assumption that one or more species must be at low abundance levels.

Note though, that our analytical arguments revealed a necessary requirement for feasibility is that$\boldsymbol{\gamma}\ll1$ (eqn.13), which permits smaller *spread.* In the extreme situation just before feasibility is lost, the smallest population is anyhow small $N_{min}^{*}\ll1$, so that $spread\simeq1>\boldsymbol{\gamma}$**.** As such, the approximations should generally be valid for feasible systems as numerical tests corroborate. Approximation eqn.13 is accurate in many scenarios even if some species are *not* at relatively low population levels (see Fig.S2).

The relationship is ultimately dependent on the competition between two factors (a) the effects of the diagonal matrix **D***=diag(*$N_{i}^{*}$*)* including the variability of the populations$N_{i}^{*}$, and (b) the strength γ of the off-diagonal perturbations. Relationship eqn.13 will hold, if the influence of the diagonal dominates the influence of the noise.

Thus there are two regimes to consider for which the eigenvalue distributions of **S** in the complex plane are qualitatively different. First, without loss of generality, we rescale all equilibrium populations so that $N_{max}^{*}=1.$ The two regimes are:

***i)*** γ $\gtrsim$ *G =* $N_{max}^{*}-N_{min}^{*}$ . In this regime, the variability in the off-diagonal interaction perturbations $\gamma$ is large compared to the variability of the equilibrium populations. For example, in the extreme case, where all population equilibria are identical, say $N_{i}^{*}=1$, and their variability is zero (*G*=0), then the community matrix $\mathbf{S=DA=A}.$ In this situation, we return to May’s analysis which predicts $\Lambda\simeq-\left( 1- \gamma\right),$ and that the eigenvalue distribution of **S** follows the semi-circle law.

***ii)*** γ $\lesssim$*G*. In the second regime, the variability in the noise $\gamma$ is smaller or of the same order as the variability in the equilibrium populations. Under these conditions the impact of the diagonal matrix **D***=diag(*$N_{i}^{*}$) overpowers the off-diagonal perturbations resulting in$\Lambda\simeq{-N}_{min}^{*}$ (eg., as seen easily for the case $\gamma=0$), as described in the main text.

Through numerical exploration we have found there is an expected threshold that defines a switch between the two regimes *i* and *ii*.

This is illustrated in Fig.S3. which provides a set of 122 graphs of the eigenvalue distributions of **S=DA** in the complex plane for 122 different parameter values. Going vertically down the figure, graphs of increasing γ are plotted, with γ varying from 0.01 to 1. Moving from left to right changes *G* from unity to zero. The thick green ray indicates the approximate threshold line γ=*G*.

All the graphs for which γ $\lesssim$ *G*  (the graphs above the green diagonal) belong to regime *ii*, where the relationship:$\Lambda\simeq{-N}_{min}^{*}$, holds*.* In these graphs the blue symbol + indicates${-N}_{min}^{*}$, and it is found at the right most end on the eigenvalue distribution almost exactly where $\Lambda$ is located. The eigenvalue distribution is wedged between $[{-N}_{max}^{*}$, ${-N}_{min}^{*}]$ on the real axis.

All the graphs for which γ $\gtrsim$ *G*  (the graphs below the green diagonal) belong to regime *i*, where May’s semi-circle law kicks in because of the relatively large interaction perturbations swamping the effects of the diagonal dominance*.* Now the blue symbol + indicates that${-N}_{min}^{*}$, is no longer found at the right most edge of the eigenvalue distribution where $\Lambda$ is located.

Each graph in Figure S3 plots the eigenvalue distribution of **S=DA** in the complex plane for *n*=200 species. Random population equilibria$N_{i}^{*}$ are drawn from a uniform distribution in the interval (1-G,1). The left hand axis of the figure specifies the value of γ while the top right hand axis specifies the value of *G* used to create any given graph. Thus G=1 implies we are selecting *n* random population equilibria $N_{i}^{*}$from the interval (0,1).


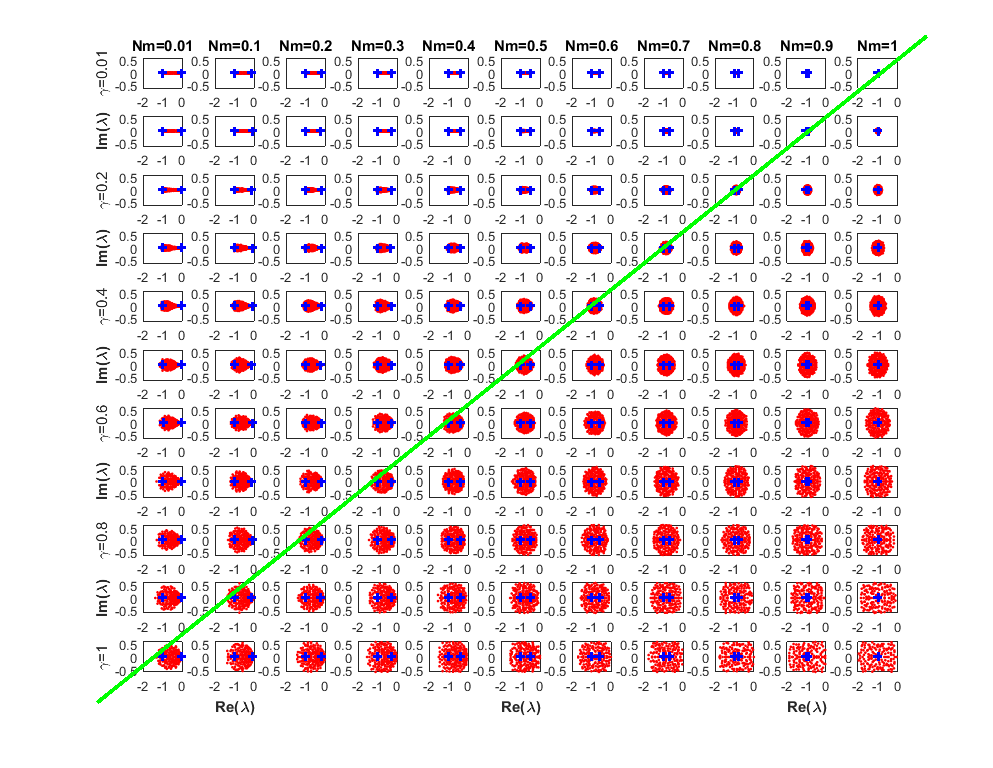


**Figure S3**  The general density dependent model. 122 plots of eigenvalues of S in complex plane for 122 different parameters G and γ when n=200. Horizontal axis is in terms of ${G=N_{max}^{*}-N}_{min}^{*}$ , , where G varies from G=1 (LHS) to G=0 (RHS). Vertical axis gives γ varying from γ=0 to γ=1.

To test the prediction eqn.13 of the main text that $\Lambda={-N}_{min}^{*}$, we plot the error of the prediction over a large parameter space for γ and for ${Nm=N}_{min}^{*}$.

Because population equilibrium numbers range in the (0,1) interval, and often attain low levels ${(N}_{min}^{*}\ll1)$ , a definition of error in this context was taken to be absolute error expressed as a percentage of $N_{max}^{*}=1.$ $i.e.,$ E=(-$\Lambda{-N}_{min}^{*})*100$ % .


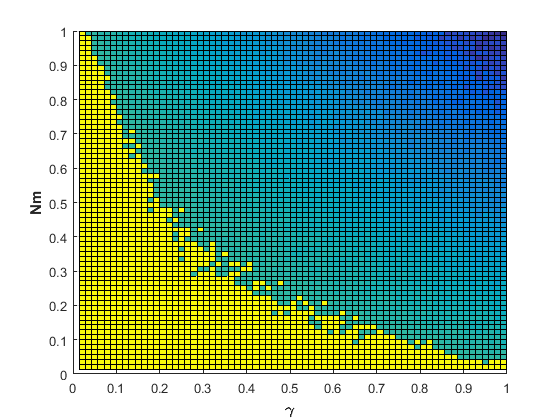

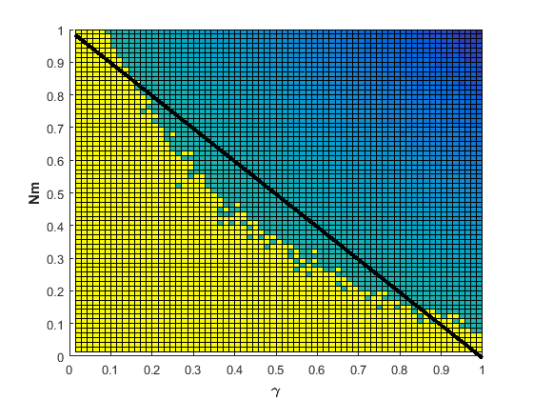


**Figure S4** Prediction error that visualises how well ${-N}_{min}^{*}$ estimates.$\Lambda$..

LH panel E<2%., and RH panel E<6% as plotted in bright yellow.

The figure S4 plots E=${(-N}_{min}^{*}$-$\Lambda$ )*100 which is zero when prediction eqn.8 holds and ${-N}_{min}^{*}= \Lambda$. Bright yellow indicates prediction error of less than 2% i.e., E<2%. in the LH panel and E<6% in RH panel. .The black diagonal line highlights the line G= γ, a rough approximation for regime shift as discussed above.

**SI5 Resiliency of competition versus mutualist communities: A comparison of methods** The “great god of competition” concept (Simberloff 1984) has been a long-held principle amongst ecologists, for which competition is viewed as the main stabilizing force in ecological communities, while cooperation is viewed as essentially unstable. It is interesting to re-examine this principle by studying the stability properties of the interaction matrix **A**, and comparing with conclusions based on the community matrix **S=DA**.

**i) Community matrix S=A, indicates mutualists are destabilizing .**

Based on the RMT system suppose the Jacobian **S** is defined as

$s_{ij}= {N_{i}^{*}a}_{ij}=\left( m+b_{ij} \right)>0, s_{ii}=-1$ and $m>0, \left| b_{ij} \right|<m.$

An underlying and unstated assumption is that all equilibrium populations are scaled to unity $N_{i}^{*}=1$, which effectively means we need only study the stability characteristics of the interaction matrix **A** with $s_{ij}=a_{ij}$. Thus from the outset, the framework assumes that a feasible equilibrium exists, which may be a wrong assumption.

The critical eigenvalue $\Lambda$ = $\max_{i} {Re(}_{i})$ of **S=A**, for these mutualistic systems may be approximated (for large *n*) by the row sum of **S***,* namely $\Lambda\cong-1+\left( n-1 \right)m$. In short, $\Lambda$ increases with *m*, and the feasible equilibrium becomes less and less resilient as *m* increases. A sufficient condition for instability of the equilibrium is that $m>1/(n-1)$, when the uniform model loses stability.

**ii)** **Community matrix S=DA indicates mutualists have no effect or a positive effect on resilience.**

Using the more traditional approach of directly perturbing the species-interactions, as advocated here, the community matrix then has elements:

$s_{ij}= {N_{i}^{*}a}_{ij}=N_{i}^{*} (m+b_{ij}), s_{ii}=-N_{i}^{*}$ and $m>0.$

The equilibrium $N_{i}^{*}$are solutions of the the LV model (eqns.14) whereby **AN*=-1**. Thus the community matrix **SN*= -1 N***, has an eigenvalue of -1 (see Methods). This “outlier” eigenvalue is well separated from the “bulk” as shown in Fig.S5. The critical eigenvalue of **S** proves to be $\Lambda=-1$ for all values$m$ for which there is a feasible equilibrium. Thus the degree of mutualistic interaction *m* has *no* impact on the resiliency of a feasible equilibrium. If the average eigenvalue is used as an index to gauge resiliency of a feasible equilibrium, it is possible to show that the strength of mutualistic interactions *m* significantly increases the resiliency of feasible systems.

The above analysis makes clear that the two different viewpoints **S=A** versus **S=DA** can lead to very different conclusions about resiliency. The study of the true community matrix **S=DA** (the method advocated here), finds mutualism should not be viewed as a destabilising process, and can often be stabilising in terms of resiliency, while competition is destabilising.

Competition systems on the other hand appears destabilizing, despite the “great god of competition” because increasing *c* increases the critical eigenvalue $\Lambda$ = $\max_{i} {Re(}_{i})=-\left( 1-c \right)+\sqrt{n}\sigma,$ of **A** and also the critical eigenvalue of **S=DA** thereby decreasing resiliency

**SI6: The effects of correlations between D and S=DA**

To determine the eigenvalue distribution of the stability matrix **S=DA** from the matrix **A**, we have used a result from Ahmadian et al (2015). However this particular result assumes the matrices **D** and **A** are independent. In our case $\mathbf{D}=-diag\left( N_{i}^{*} \right),$ and the equilibrium populations $N_{i}^{*}$ are dependent on the entries of the matrix **A**. Thus the matrices **D** and **A** will be correlated to some extent.

In fact the paper of Ahmadian et al. (2015) considered a similar problem, an example of a classical neuron system which studies the same form of the stability matrix **S=DA** where$\mathbf{D}=-diag\left( N_{i}^{*} \right).$ Their analysis thus suffers from a similar dependency problem, which they addressed as follows:

“In closing we note a potential caveat in the applicability of our formulae …. We have derived the general formulae …. assuming that M, L and R are independent of J. However, M and R …. depend on J via their dependence on [$N_{i}^{*}$]. However, in our experience this dependence is often too weak and indirect to render our formulae inapplicable; an example is provided by the excellent agreement of the empirical spectrum …. which also held for other parameter choices of the model of Ref. [48].”

In the May model studied here, the problem mentioned in caveat appears to be also relatively minor. That is, the “dependence is often too weak and indirect to render our formulae inapplicable”. This is because the LV system loses feasibility for$\gamma\ll1$. The stability of **S=DA** is lost exactly when feasibility is lost, namely for$\gamma\ll1.$ However, in this regime variability is relatively small ensuring the effects of dependence and correlation are relatively small and have little impact. This is seen in the following three plots of Fig.S6 for a) relatively small $\gamma=0.1$; b) intermediate $\gamma=0.4$, where $\gamma$ is large enough that feasibility is at the point of being lost. c) very large $\gamma=0.9,$ where some equilibrium populations are negative and the matrix **S=DA** is unstable. In all three cases the “bulk” eigenvalue boundary predicted by Eqn.9 appears to be accurate.

1. γ **=0.1**

**
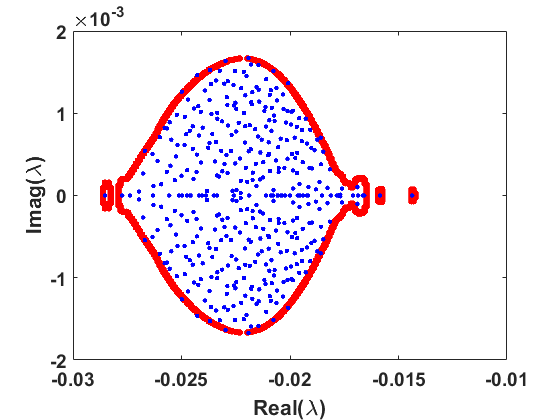
**

1. **
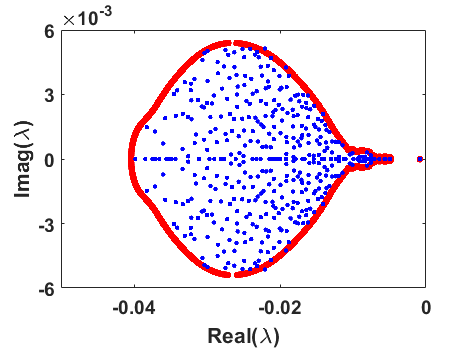
**γ **=0.4**
2. γ **=0.9**

**
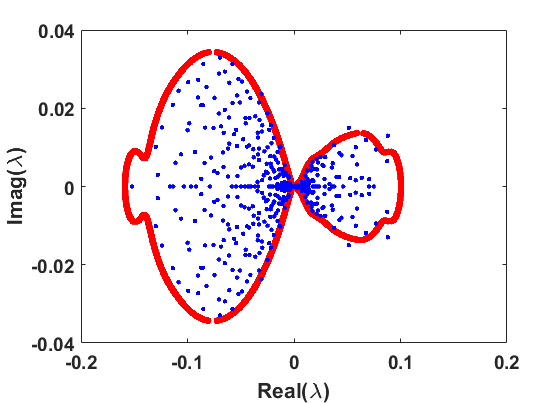
**

**Fig. S5** Competition community with *n=400, c=0.1* a) $\gamma$ *=0.1;* b) $\gamma$ *=0.4;* c) $\gamma$ *=0.9* illustrating that the boundary of the eigenvalues (blue dots) of **S=DA** is predicted well by Eqn.16 (red line). This is despite the correlations between the matrices **D** and **A** which are expected to become larger as $\gamma$ increases.

**Additional References**

1. Kokkoris, G.D., Jansen, V.A.A., Loreau, M. & Troumbis, A.Y. Variability in interaction strength and implications for biodiversity. *Journal of Animal Ecology* 71, 362-371. (2002).
2. Pomerantz, M.J. & Gilpin, M.E. Community covariance and coexistence. *Journal of Theoretical Biology* 79, 67-81 (1979).
3. Roberts, A. When will a large complex system be viable? Environmental Discussion Paper, Graduate School of Environmental Science, Monash University, Melbourne, Australia. (1989).
4. Stone, L. & Roberts, A. Conditions for a species to gain advantage from the presence of competitors.  *Ecology,* 1964-1972 (1991).
5. Wigner, E.P. Random matrices in physics. *SIAM Review* 9:1-23. (1967).
6. M.J. Manton On feasible random ecosystems. Bulletin Math Biology 41:751-755 (1979)
7. G.H. Golub, C.F. Van Loan. Matrix Computations. The John Hopkins University Press. (2012)
8. F. Benaych-Georges, N. Enriquez, A. Michail. Perturbations by random matrices. Arxiv:1701.02597 v2
9. R. Allez, J. Bun, J.-P. Bouchaud. The eigenvectors of Gaussian matrices with an external source. *arXiv:1412.7108* (2014).
10. Y. Shmueli, G. Wolf, A. Averbuch. Updating kernel methods in spectral decomposition by affinity perturbations. Linear Algebra and its Applications. 437:356-1365 (2012)
11. M.J. Manton On feasible random ecosystems. Bulletin Math Biology 41:751-755 (1979)
12. Rozdilsky, I. and Stone L. Complexity can Enhance Stability in Competitive systems. Ecology Letters 4:397-400. 2001.
13. Simberloff, D. The great god of competition. *The Sciences* **24**, 17-22 (1984).
